# Supplementary material for: Analyzing first aid in textbooks used by non-medical and paramedical students in Nepal: A need of further attention for snakebite management!
Source: PLoS Negl Trop Dis. 2025 Dec 2;19(12):e0013765. doi: 10.1371/journal.pntd.0013765 (PMC12680362; doi:10.1371/journal.pntd.0013765)
Supplement: S1 Table — (DOCX) [file pntd.0013765.s001.docx]

| **S1 Table.** Nepalese universities, associated institutions, and total students enrollment yearly in Bachelor's Degree in Health/Nursing in which curriculum includes first aid of snakebite. | | | | |
| --- | --- | --- | --- | --- |
| **University, Institution, address** | | **Courses** | **Seats** | **Web links** |
| TU | Maharajgunj Medical Campus Maharajgunj, Kathmandu | Bachelor of Dental Surgery (BDS) | 25 | # https://edusanjal.com/college/maharajgunj-medical-campus/ |
| TU | Maharajgunj Medical Campus Maharajgunj, Kathmandu | Bachelor of Pharmacy (B. Pharm) | 20 | # https://edusanjal.com/college/maharajgunj-medical-campus/ |
| TU | People's Dental College Balaju, Kathmandu | Bachelor of Public Health (BPH) | 20 | # https://edusanjal.com/college/peoples-dental-college/ |
| TU | People's Dental College Balaju, Kathmandu | Bachelor of Dental Surgery (BDS) | 50 | # https://edusanjal.com/college/peoples-dental-college/ |
| TU | National Medical College Birgunj, Parsa | Bachelor of Dental Surgery (BDS) | 15 | # https://edusanjal.com/college/national-medical-college/ |
| TU | National Medical College Birgunj, Parsa | Bachelor of Nursing Science (BNS) | 20 | # https://edusanjal.com/college/national-medical-college/ |
| TU | National Medical College Birgunj, Parsa | B.Sc. Nursing | 20 | # https://edusanjal.com/college/national-medical-college/ |
| TU | MB Kedia Dental College and Teaching Hospital Birgunj, Birgunj, Parsa | Bachelor of Dental Surgery (BDS) | 3 | # https://edusanjal.com/college/national-medical-college/ |
| TU | Chitwan Medical College Bharatpur, Chitwan | Bachelor of Dental Surgery (BDS) | 40 | # https://edusanjal.com/college/chitwan-medical-college/ |
| TU | Chitwan Medical College Bharatpur, Chitwan | Bachelor of Nursing Science (BNS) | 40 | # https://edusanjal.com/college/chitwan-medical-college/ |
| TU | Chitwan Medical College Bharatpur, Chitwan | B.Sc. Nursing | 40 | # https://edusanjal.com/college/chitwan-medical-college/ |
| TU | Chitwan Medical College Bharatpur, Chitwan | Bachelor of Pharmacy (B. Pharm) | 40 | # https://edusanjal.com/college/chitwan-medical-college/ |
| TU | Chitwan Medical College Bharatpur, Chitwan | Bachelor of Public Health (BPH) | 40 | # https://edusanjal.com/college/chitwan-medical-college/ |
| TU | Gandaki Medical College Gandaki Medical College Lekhanath, Pokhara, Kaski | Bachelor of Dental Surgery (BDS) | 30 | # https://edusanjal.com/college/gandaki-medical-college/ |
| TU | Gandaki Medical College Gandaki Medical College Lekhanath, Pokhara, Kaski | Bachelor of Public Health (BPH) | 20 | # https://edusanjal.com/college/gandaki-medical-college/ |
| TU | Gandaki Medical College Gandaki Medical College Lekhanath, Pokhara, Kaski | Bachelor of Nursing Science (BNS) | 20 | # https://edusanjal.com/college/gandaki-medical-college/ |
| TU | Gandaki Medical College Gandaki Medical College Lekhanath, Pokhara, Kaski | B.Sc. Nursing | 40 | # https://edusanjal.com/college/gandaki-medical-college/ |
| TU | Gandaki Medical College Gandaki Medical College Lekhanath, Pokhara, Kaski | Bachelor of Pharmacy (B. Pharm) | 10 | # https://edusanjal.com/college/gandaki-medical-college/ |
| TU | KIST Medical College Gwarko, Imadol, Lalitpur | Bachelor of Pharmacy (B. Pharm) | 10 | # https://edusanjal.com/college/kist-medical-college/ |
| TU | KIST Medical College Gwarko, Imadol, Lalitpur | Bachelor of Dental Surgery (BDS) | 40 | # https://edusanjal.com/college/kist-medical-college/ |
| TU | Universal College of Medical Sciences and Teaching Hospital Bhairahawa, Siddharthanagar, Rupandehi | Bachelor of Dental Surgery (BDS) | 50 | # https://edusanjal.com/college/universal-college-medical-sciences-and-teching-hospital/ |
| TU | Universal College of Medical Sciences and Teaching Hospital Bhairahawa, Siddharthanagar, Rupandehi | Bachelor of Nursing Science (BNS) | 40 | # https://edusanjal.com/college/universal-college-medical-sciences-and-teching-hospital/ |
| TU | Universal College of Medical Sciences and Teaching HospitalBhairahawa, Siddharthanagar, Rupandehi | Bachelor of Public Health (BPH) | 20 | # https://edusanjal.com/college/universal-college-medical-sciences-and-teching-hospital/ |
| TU | Universal College of Medical Sciences and Teaching Hospital Bhairahawa, Siddharthanagar, Rupandehi | B.Sc. Nursing | 40 | # https://edusanjal.com/college/universal-college-medical-sciences-and-teching-hospital/ |
| TU | Universal College of Medical Sciences and Teaching Hospital Bhairahawa, Siddharthanagar, Rupandehi | Bachelor of Pharmacy (B. Pharm) | 40 | # https://edusanjal.com/college/universal-college-medical-sciences-and-teching-hospital/ |
| TU | Nepal Ayurved Medical College and Teaching Hospital Birgunj, Parsa | Bachelor of Ayurvedic Medicine and Surgery (BAMS) | 78 | # https://edusanjal.com/college/nepal-ayurved-medical-college/ (No "seats" mentioned); https://www.educatenepal.com/institutions/detail/nepal-ayurved-medical-college |
| TU | Ayurveda Campus Kirtipur, Kathmandu | Bachelor of Ayurvedic Medicine and Surgery (BAMS) | 40 | # https://edusanjal.com/college/ayurveda-campus/ |
| NSU | Central Ayurveda Vidyapeeth Bijauri, Tulsipur, Dang | Bachelor of Ayurvedic Medicine and Surgery (BAMS) | 40 | # https://edusanjal.com/college/central-ayurveda-vidyapeetha/ |
| NSU | Patanjali Ayurved Medical College and Research Center Dhulikhel, Kavrepalanchok | Bachelor of Ayurvedic Medicine and Surgery (BAMS) | 20 | # https://edusanjal.com/college/patanjali-ayurved-medical-college-and-research-center/ |
| NSU | Shree Mithila Ayurveda College and Research Centre Nagarain – 5, Dhanusha, Nagarain, Nagarain, Dhanusha | Bachelor of Ayurvedic Medicine and Surgery (BAMS) | NA | # https://edusanjal.com/college/mithila-ayurveda-college-and-research-center/ (No "seats" mentioned); |
| KU | Kantipur Dental College Basundhara, Kathmandu | Bachelor of Dental Surgery (BDS) | 20 | # https://edusanjal.com/college/kantipur-dental-college/ |
| KU | Nepal Medical College Attarkhel, Jorpati, Gokarneshwar, Kathmandu | Bachelor of Dental Surgery (BDS) | 45 | # https://edusanjal.com/college/nepal-medical-college/ |
| KU | Nepal Medical College Attarkhel, Jorpati, Gokarneshwar, Kathmandu | B.Sc. Nursing | 40 | # https://edusanjal.com/college/nepal-medical-college/ |
| KU | Kathmandu Medical College Sinamangal, Kathmandu | Bachelor of Dental Surgery (BDS) | 50 | # https://edusanjal.com/college/kathmandu-medical-college/ |
| KU | Kathmandu Medical College Sinamangal, Kathmandu | Bachelor of Nursing Science (BNS) | 20 | # https://edusanjal.com/college/kathmandu-medical-college/ |
| KU | Kathmandu Medical College Sinamangal, Kathmandu | B.Sc. Nursing | 40 | # https://edusanjal.com/college/kathmandu-medical-college/ |
| KU | Nepalgunj Medical College Nepalgunj, Kohalpur, Banke | Bachelor of Dental Surgery (BDS) | 15 | # https://edusanjal.com/college/nepalgung-medical-college/ |
| KU | Nepalgunj Medical College Nepalgunj, Kohalpur, Banke | B.Sc. Nursing | 20 | # https://edusanjal.com/college/nepalgung-medical-college/ |
| KU | College of Medical Sciences Teaching Hospital Bharatpur, Chitwan | Bachelor of Dental Surgery (BDS) | 45 | # https://edusanjal.com/college/college-medical-sciences/ |
| KU | College of Medical Sciences Teaching Hospital Bharatpur, Chitwan | B.Sc. Nursing | 40 | # https://edusanjal.com/college/college-medical-sciences/ |
| KU | Kathmandu University School of Medical Sciences Dhulikhel, Kavrepalanchok | Bachelor of Nursing Science (BNS) | 30 | # https://edusanjal.com/college/kathmandu-university-school-medical-science/ |
| KU | Kathmandu University School of Medical Sciences Dhulikhel, Kavrepalanchok | B.Sc. Nursing | 40 | # https://edusanjal.com/college/kathmandu-university-school-medical-science/ |
| KU | Nobel Medical CollegeBiratnagar, Morang | Bachelor of Nursing Science (BNS) | 20 | # https://edusanjal.com/college/nobel-medical-college/ |
| KU | Nobel Medical College Biratnagar, Morang | B.Sc. Nursing | 20 | # https://edusanjal.com/college/nobel-medical-college/ |
| KU | Nobel Medical College Biratnagar, Morang | Bachelor of Dental Surgery (BDS) | 30 | # https://edusanjal.com/college/nobel-medical-college/ |
| PoU | School of Health and Allied Sciences \| Pokhara University Dhungepatan, Pokhara, Kaski | Bachelor of Public Health (BPH) | 40 | # https://edusanjal.com/college/pokhara-university-school-of-health-and-allied-sciences/ |
| PoU | School of Health and Allied Sciences \| Pokhara University Dhungepatan, Pokhara, Kaski | B.Sc. Nursing | 20 | # https://edusanjal.com/college/pokhara-university-school-of-health-and-allied-sciences/ |
| PoU | School of Health and Allied Sciences \| Pokhara University Dhungepatan, Pokhara, Kaski | Bachelor of Pharmacy (B. Pharm) | 40 | # https://edusanjal.com/college/pokhara-university-school-of-health-and-allied-sciences/ |
| PoU | CIST College Sangam Chowk, New Baneshwor, Kathmandu | Bachelor of Pharmacy (B. Pharm) | 40 | # https://edusanjal.com/college/cist-college/ |
| PoU | CIST College Sangam Chowk, New Baneshwor, Kathmandu | Bachelor of Public Health (BPH) | 40 | # https://edusanjal.com/college/cist-college/ |
| PoU | Nobel College Sinamangal, Kathmandu | Bachelor of Pharmacy (B. Pharm) | 40 | # https://edusanjal.com/college/nobel-college/ |
| PoU | Nobel College Sinamangal, Kathmandu | Bachelor of Public Health (BPH) | 40 | # https://edusanjal.com/college/nobel-college/ |
| PoU | Nobel College Sinamangal, Kathmandu | B.Sc. Nursing | 20 | # https://edusanjal.com/college/nobel-college/ |
| PoU | Modern Technical College Sanepa, Lalitpur | Bachelor of Pharmacy (B. Pharm) | 20 | # https://edusanjal.com/college/modern-technical-college/ |
| PoU | Modern Technical College Sanepa, Lalitpur | Bachelor of Public Health (BPH) | 20 | # https://edusanjal.com/college/modern-technical-college/ |
| PoU | National Open College Sanepa, Ring Road ( Near Star Hospital), Lalitpur | Bachelor of Public Health (BPH) | 20 | # https://edusanjal.com/college/national-open-college/ |
| PoU | Crimson College of Technology Devinagar, Butwal, Rupandehi | Bachelor of Public Health (BPH) | 10 | # https://edusanjal.com/college/crimson-college-technology/ |
| PoU | Crimson College of Technology Devinagar, Butwal, Rupandehi | Bachelor of Pharmacy (B. Pharm) | 30 | # https://edusanjal.com/college/crimson-college-technology/ |
| PU | Little Buddha College of Health Science, Min Bhawan, Kathmandu | Bachelor of Public Health (BPH) | 20 | # https://edusanjal.com/college/little-buddha-college-health-science/#programs |
| PU | Little Buddha College of Health Science, Min Bhawan, Kathmandu | Bachelor of Pharmacy (B. Pharm) | 20 | # https://edusanjal.com/college/little-buddha-college-health-science/#programs |
| PU | Kathmandu Multiple College Gaushala, Kathmandu | Bachelor of Public Health (BPH) | 20 | # https://edusanjal.com/college/karnali-college-health-science/ |
| PU | Kathmandu Multiple College Gaushala, Kathmandu | Bachelor of Pharmacy (B. Pharm) | 15 | # https://edusanjal.com/college/karnali-college-health-science/ |
| PU | Hope International College Satdobato, Lalitpur | Bachelor of Public Health (BPH) | 20 | # https://edusanjal.com/college/hope-international-college/ |
| PU | Hope International College Satdobato, Lalitpur | Bachelor of Pharmacy (B. Pharm) | 30 | # https://edusanjal.com/college/hope-international-college/ |
| PU | Hope International College Satdobato, Lalitpur | B.Sc. Nursing | 20 | # https://edusanjal.com/college/hope-international-college/ |
| PU | Hope International College Satdobato, Lalitpur | Bachelor of Nursing Science (BNS) | 20 | # https://edusanjal.com/college/hope-international-college/ |
| PU | Everest College of Nursing Tinkune, Subidhanagar, Kathmandu | B.Sc. Nursing | 20 | # https://edusanjal.com/college/everest-college-nursing/ |
| PU | Everest College of Nursing Tinkune, Subidhanagar, Kathmandu | Bachelor of Nursing Science (BNS) | 20 | # https://edusanjal.com/college/everest-college-nursing/ |
| PU | Novel Academy New road, Pokhara, Kaski | Bachelor of Pharmacy (B. Pharm) | 10 | # https://edusanjal.com/college/novel-academy/ |
| PU | Charak Academy, PokharaPokhara, Kaski | B.Sc. Nursing | 20 | # https://edusanjal.com/college/charak-hospital-nursing-college/ |
| PU | Charak Academy, Pokhara Pokhara, Kaski | Bachelor of Nursing Science (BNS) | 20 | # https://edusanjal.com/college/charak-hospital-nursing-college/ |
| PU | Unique Medical College & Teaching Hospital Rajbiraj-7, Rajbiraj, Saptari | Bachelor of Public Health (BPH) | 10 | # https://edusanjal.com/college/unique-college-medical-science/ |
| PU | Norvic Institute of Nursing Education Maharajgunj, Kathmandu | B.Sc. Nursing | 20 | # https://edusanjal.com/college/norvic-institute-nursing-education/ |
| PU | Norvic Institute of Nursing Education Maharajgunj, Kathmandu | Bachelor of Nursing Science (BNS) | 40 | # https://edusanjal.com/college/norvic-institute-nursing-education/ |
| PU | Chakrabarti Habi Education Academy (College of Allied Health Science) Madhyapur Thimi, Madhyapur Thimi, Bhaktapur | Bachelor of Public Health (BPH) | 10 | # https://edusanjal.com/college/chakrabarti-habi-education-academy-college-allied-health-science/ |
| PU | SANN Institute of Nursing Khusibu, Kathmandu | B.Sc. Nursing | 20 | # https://edusanjal.com/college/sann-institute-nursing/ |
| PU | Kathmandu Model Hospital School of Nursing Thulo Bharyang, Kathmandu | Bachelor of Nursing Science (BNS) | 40 | # https://edusanjal.com/college/kathmandu-model-hospital-school-nursing/ |
| PU | Kathmandu Model Hospital School of Nursing Thulo Bharyang, Kathmandu | B.Sc. Nursing | 20 | # https://edusanjal.com/college/kathmandu-model-hospital-school-nursing/ |
| PU | Nepal Institute of Health Sciences (Stupa College) Jorpati, Gokarneshwar, Kathmandu | Bachelor of Public Health (BPH) | 20 | # https://edusanjal.com/college/nepal-institute-health-sciences/ |
| PU | B and B Medical Institute Gwarko, Lalitpur | Bachelor of Nursing Science (BNS) | 40 | # https://edusanjal.com/college/b-and-b-medical-institute-nursing-college/ |
| PU | B and B Medical Institute Gwarko, Lalitpur | B.Sc. Nursing | 20 | # https://edusanjal.com/college/b-and-b-medical-institute-nursing-college/ |
| PU | Shree Medical and Technical College Bharatpur, Chitwan | Bachelor of Nursing Science (BNS) | 40 | # https://edusanjal.com/college/shree-medical-and-technical-college/ |
| PU | Shree Medical and Technical College Bharatpur, Chitwan | B.Sc. Nursing | 20 | # https://edusanjal.com/college/shree-medical-and-technical-college/ |
| PU | Shree Medical and Technical College Bharatpur, Chitwan | Bachelor of Public Health (BPH) | 20 | # https://edusanjal.com/college/shree-medical-and-technical-college/ |
| PU | Shree Medical and Technical College Bharatpur, Chitwan | Bachelor of Pharmacy (B. Pharm) | 30 | # https://edusanjal.com/college/shree-medical-and-technical-college/ |
| PU | Hamro School of Nursing Biratnagar, Morang | B.Sc. Nursing | 20 | # https://edusanjal.com/college/hamro-school-nursing/ |
| PU | Yeti Health Science Academy Kantimarga, Maharajgunj, Kathmandu | Bachelor of Nursing Science (BNS) | 20 | # https://edusanjal.com/college/yeti-health-science-academy/ |
| PU | Yeti Health Science Academy Kantimarga, Maharajgunj, Kathmandu | B.Sc. Nursing | 20 | # https://edusanjal.com/college/yeti-health-science-academy/ |
| PU | Yeti Health Science Academy Kantimarga, Maharajgunj, Kathmandu | Bachelor of Public Health (BPH) | 20 | # https://edusanjal.com/college/yeti-health-science-academy/ |
| PU | Nepal Polytechnic Institute Bharatpur, Chitwan | B.Sc. Nursing | 20 | # https://edusanjal.com/college/nepal-polytechnic-institute/ |
| PU | Oasis Medical College Bharatpur, Chitwan | Bachelor of Public Health (BPH) | 10 | # https://edusanjal.com/college/oasis-medical-college/ |
| PU | NAMS CollegeOld Baneshwor, Kathmandu | Bachelor of Public Health (BPH) | 10 | # https://edusanjal.com/college/nams-college/ |
| PU | NAMS College Old Baneshwor, Kathmandu | B.Sc. Nursing | 20 | # https://edusanjal.com/college/nams-college/ |
| PU | Om Health Campus Chabahil, Kathmandu | Bachelor of Nursing Science (BNS) | 40 | # https://edusanjal.com/college/om-health-campus/ |
| PU | Om Health Campus Chabahil, Kathmandu | B.Sc. Nursing | 20 | # https://edusanjal.com/college/om-health-campus/ |
| PU | Om Health Campus Chabahil, Kathmandu | Bachelor of Public Health (BPH) | 20 | # https://edusanjal.com/college/om-health-campus/ |
| PU | Nagarik College of Health Sciences Koteshwor, Kathmandu | Bachelor of Nursing Science (BNS) | 40 | # https://edusanjal.com/college/nagarik-college-health-sciences/ |
| PU | Nagarik College of Health Sciences Koteshwor, Kathmandu | B.Sc. Nursing | 20 | # https://edusanjal.com/college/nagarik-college-health-sciences/ |
| PU | Krishna Medical and Technical Research Centre Janakpur, Janakpurdham, Dhanusha | Bachelor of Nursing Science (BNS) | 20 | # https://edusanjal.com/college/krishna-medical-and-technical-research-centre/ |
| PU | Krishna Medical and Technical Research Centre Janakpur, Janakpurdham, Dhanusha | B.Sc. Nursing | 20 | # https://edusanjal.com/college/krishna-medical-and-technical-research-centre/ |
| PU | Kantipur Academy of Health Science Tinkune, Kathmandu | Bachelor of Public Health (BPH) | 10 | # https://edusanjal.com/college/kantipur-academy-institute-of-health-science/ |
| PU | Kantipur Academy of Health Science Tinkune, Kathmandu | B.Sc. Nursing | 20 | # https://edusanjal.com/college/kantipur-academy-institute-of-health-science/ |
| PU | Kantipur Academy of Health Science Tinkune, Kathmandu | Bachelor of Nursing Science (BNS) | 20 | # https://edusanjal.com/college/kantipur-academy-institute-of-health-science/ |
| PU | Kantipur Academy of Health Science Tinkune, Kathmandu | Bachelor of Pharmacy (B. Pharm) | 10 | # https://edusanjal.com/college/kantipur-academy-institute-of-health-science/ |
| PU | Valley College of Technical Sciences Sitapaila-2, Kathmandu | Bachelor of Pharmacy (B. Pharm) | 30 | # https://edusanjal.com/college/valley-college-technical-sciences/ |
| PU | Valley College of Technical Sciences Sitapaila-2, Kathmandu | Bachelor of Public Health (BPH) | 10 | # https://edusanjal.com/college/valley-college-technical-sciences/ |
| PU | Sanjeevani College of Medical Sciences (Sanjeevani Nursing College) Kalika Nagar, Butwal, Rupandehi | Bachelor of Public Health (BPH) | 10 | # https://edusanjal.com/college/sanjeevani-nursing-college/ |
| PU | Asian College for Advance Studies Satdobato, Lalitpur | Bachelor of Pharmacy (B. Pharm) | 30 | # https://edusanjal.com/college/asian-college-advance-studies/ |
| PU | Asian College for Advance Studies Satdobato, Lalitpur | Bachelor of Public Health (BPH) | 20 | # https://edusanjal.com/college/asian-college-advance-studies/ |
| PU | Asian College for Advance Studies Satdobato, Lalitpur | Bachelor of Nursing Science (BNS) | 20 | # https://edusanjal.com/college/asian-college-advance-studies/ |
| PU | Asian College for Advance Studies Satdobato, Lalitpur | B.Sc. Nursing | 20 | # https://edusanjal.com/college/asian-college-advance-studies/ |
| PU | Purbanchal University School of Health Sciences Gothgaun, Sundarharaicha, Morang | Bachelor of Pharmacy (B. Pharm) | 30 | # https://edusanjal.com/college/purbanchal-university-college-medical-and-allied-sciences/ |
| PU | Purbanchal University School of Health Sciences Gothgaun, Sundarharaicha, Morang | Bachelor of Public Health (BPH) | 40 | # https://edusanjal.com/college/purbanchal-university-college-medical-and-allied-sciences/ |
| PU | Purbanchal University School of Health SciencesGothgaun, Sundarharaicha, Morang | Bachelor of Nursing Science (BNS) | 40 | # https://edusanjal.com/college/purbanchal-university-college-medical-and-allied-sciences/ |
| PU | Purbanchal University School of Health Sciences Gothgaun, Sundarharaicha, Morang | B.Sc. Nursing | 40 | # https://edusanjal.com/college/purbanchal-university-college-medical-and-allied-sciences/ |
|  | **Total** |  | **3041** |  |
| **Abbreviations:** **TU** stands for Tribhuvan University, **NSU**: Nepal Sanskrit University, **KU**: Kathmandu University, **PoU**: Pokhara University, **PU**: Purbanchal University | | | | |
