# Supplementary material for: Analyzing first aid in textbooks used by non-medical and paramedical students in Nepal: A need of further attention for snakebite management!
Source: PLoS Negl Trop Dis. 2025 Dec 2;19(12):e0013765. doi: 10.1371/journal.pntd.0013765 (PMC12680362; doi:10.1371/journal.pntd.0013765)
Supplement: S2 Table — (DOCX) [file pntd.0013765.s002.docx]

| **S2 Table.** School and university textbooks currently used in Nepal and having potential of advising measures for first aid of snakebites. | | | | | | | | | |
| --- | --- | --- | --- | --- | --- | --- | --- | --- | --- |
| **Secondary coding** | **Primary coding of textbooks (€)** | **Classes** | **Book`s name/medium** used in book writing | **Publishers, the city and country** where the books were printed | **Publication Year** (i.e., editions and reprint years) **of books** | **Author/s** of respective textbooks | **Editor** | Containing **"Safety and First Aid"** unit | **Page ranges** devoted to snakebite health education |
| 4.1 | 4_HPE&CA_22_Eng_Baruwal_Akar_NA | 4 | Health, Physical Education and Creative Arts (HPE&CA)*****/ English | Aakar Pubisher & Distributor Pvt. Ltd. (APD PL), Kathmandu (Ktm) | First edition: 2079 BS (2022 AD); Reprint: 2080,81 BS (2023, 24 AD) | Arjun K. Baruwal (AKB) | NA | Yes | No first aid mentioned. |
| 4.2 | 4_HPE&CA_22_Eng_Baral_Sangam_NA | 4 | HPE&CA*****/ English | Sangam Books Publication Pvt. Ltd. (SBP PL), Ktm | First edition: 2011 AD; Reprint: 2012 AD; Revised edition: 2016 AD; Second edition: 2022 AD, Reprint: 2023 AD | Tara Nath Baral (TNB) | NA | Yes | No first aid mentioned. |
| 4.3 | 4_HP&CA_23_Nep_CDC_NA | 4 | Health, Physical, and Creative Arts (HP&CA)******/ Nepali | CDC, Sanothimi, Bhaktapur, Nepal | First edition: 2079 BS (2023 AD) | Curriculum Development Center (CDC) | NA | Yes | No first aid mentioned. |
| 4.4 | 4_HP&CA_23_Eng_CDC_NA | 4 | Health, Physical, and Creative Arts (HP&CA)******/ English | CDC, Sanothimi, Bhaktapur, Nepal | First edition: 2022 AD | CDC | NA | Yes | No first aid mentioned. |
| 5.5 | 5_HPE&CA_23_Baral_Sangam_Eng_NA | 5 | HPE&CA*****/ English | SSB PL, Ktm | First edition: 2011 AD; Reprint: 2012 AD; Revised edition: 2016 AD; Second edition: 2023 AD | TNB | Kumar Bahadur Rai (KBR) | Yes | No first aid mentioned. |
| 5.6 | 5_HPE&CA_23_Eng_Baruwal_Akar_NA | 5 | HPE&CA*****/ English | APD PL, Ktm | First edition: 2023 AD; Reprint: 2024 AD | AKB | NA | Yes | No first aid mentioned. |
| 5.7 | 5_HPE&CA_23_Eng_Aryal et al_Vidyarthi_NA | 5 | HPE&CA*****/ English | Vidyarthi Pustak Bhandar (VPB), Ktm. | First edition: 2023 AD | Dr. Bhagwan Aryal (BA), Assoc. Prof. Somnath Dhakal (SD), Nagendra Prasad Neupane (NPN) | Prof. Dr. Ram Krishna Maharjan (RKM) | Yes | No first aid mentioned. |
| 5.8 | 5_HP&CA_24_Nep_CDC_NA | 5 | Health, Physical, and Creative Arts (HP&CA)******/ Nepali | CDC, Sanothimi, Bhaktapur, Nepal | First edition: 2080 BS (2024 AD) | CDC | NA | Yes | No first aid mentioned. |
| 6.9 | 6_HPE&CA_??_Eng_Baruwal_Akar_NA | 6 | HPE&CA*****/ English | APD PL, Ktm | First editions: 2078 BS (2022 AD); Revised edition: 2024 (reprint: 2024) | AKB | NA | Yes | No first aid mentioned. |
| 6.1 | 6_HPE&CA_23_Eng_Maharjan et al_Vidhyarthi_NA | 6 | HPE&CA*****/ English | Vidyarthi Pustak Bhandar (VPB), Ktm. | First edition: 2078 BS (2022 AD), Second edition: 2079 BS (2023 AD), Third edition: 2080 BS (2023 AD) | Prof. Dr. Ram K. Maharjan (RKM), Assoc. Prof. Som N. Dhakal (SND), Dr. Bhagwan Aryal (BA), Nagendra P. Neupane (NPN) | NA | Yes | No first aid mentioned. |
| 6.11 | 6_HPE&CA_21_Eng_Kapadandi and Maharjan_Green_100 | 6 | HPE&CA*****/ English | Green Books (GB), Lalitpur | First edition: 2078 BS (2021 AD); Re-print: 2080 BS (2023 AD) | Bhan Dev Kapadi (BDK). Ram Maharjan (RM) | NA | Yes | 100 |
| 6.12 | 6_HPE&CA_24_Eng_Baral_Sangam_72-73 | 6 | HPE&CA*****/ English | Sangam Books Pubication Pvt. Ltd. (SBP PL), Kathmandu | Revised edition: 2021 AD; Revised edition: 2024 AD | TNB | Kumar Bahadur Rai (KBR) | Yes | 72-73 |
| 6.13 | 6_HP&CA_21_Eng_CDC_NA | 6 | Health, Physical, and Creative Arts (HP&CA)******/ English | CDC, Sanothimi, Bhaktapur, Nepal | First edition: 2021 AD | CDC | NA | Yes | No first aid mentioned. |
| 6.14 | 6_HP&CA_21_Nep_CDC_NA | 6 | Health, Physical, and Creative Arts (HP&CA)**/ Nepali | CDC, Sanothimi, Bhaktapur, Nepal | First edition: 2021 AD | CDC | NA | Yes | No first aid mentioned. |
| 7.15 | 7_HPE&CA_24_Eng_Baruwal_Akar_102-104 | 7 | HPE&CA*****/ English | Aakar Pubisher & Distributor Pvt. Ltd. (APD), Ktm | First edition: 2079 BS (2022 AD); Second edition: 2080 BS (2023 AD), Third edition: 2024 AD (reprint: 2024 AD) | AKB | NA | Yes | 102–104 |
| 7.16 | 7_HPE&CA_22_Eng_Aryal et al_Vidhyarthi_80-81 | 7 | HPE&CA*****/ English | Vidyarthi Pustak Bhandar Ktm. (VPB) | First edition: 2022 AD; Reprint: 2023 AD | BA, SND, NPN | Prof.Dr. Ram Krishna Maharjan (RKM) | Yes | 80–81 |
| 7.17 | 7_HPE&CA_22_Eng_Kapadi&Maharjan_Green_98 | 7 | HPE&CA*****/ English | Green Books Pubisher & Distributor Pvt. Ltd., Lalitpur (GBPD) | First edition: 2079 BS (2022 AD) Re-print: 2080 BS (2023 AD) | Bhan Dev Kapadi (BDK), Ram Maharjan (RM) | NA | Yes | 98 |
| 7.18 | 7_HPE&CA_22_Eng_Baral_Sangam_111-112 | 7 | HPE&CA*****/ English | Sangam Books Pubication Pvt. Ltd. (SBP PL), Kathmandu | First edition (**ed**): 2011 AD (reprint: 2012 AD), revised ed: 2016 AD; second ed: 2022 AD (reprint: 2023 AD, 2024 AD) | TNB | Kumar B. Rai (KBR) | Yes | 111–112 |
| 7.19 | 7_HP&CA_23_Nep_CDC_53-54 | 7 | Health, Physical, and Creative Arts (HP&CA)******/ Nepali | Curriculum Development Center (CDC), Sanothimi, Bhaktapur, Nepal | First edition: 2079 BS (2023 AD) | CDC | NA | Yes | 53–54 |
| 7.20 | 7_HP&CA_22_Eng_CDC_56-57 | 7 | Health, Physical, and Creative Arts (HP&CA)**/ English | Curriculum Development Center (CDC), Sanothimi, Bhaktapur, Nepal | First edition: 2022 AD | CDC | NA | Yes | 56–57 |
| 8.21 | 8_HP&CA_23_Eng_CDC_NA | 8 | Health, Physical, and Creative Arts (HP&CA)******/ English | CDC, Sanothimi, Bhaktapur, Nepal | First edition: 2023 AD | CDC | NA | Yes | No first aid mentioned. |
| 8.22 | 8_HP&CA_24_Nep_CDC_NA | 8 | Health, Physical, and Creative Arts (HP&CA)**/ Nepali | CDC, Sanothimi, Bhaktapur, Nepal | First edition: 2024 AD | CDC | NA | Yes | No first aid mentioned. |
| 8.23 | 8_HPE&CA_23_Eng_Baral_Sangam_NA | 8 | HPE&CA*****/ English | Sangam Books Pubication Pvt. Ltd. (SBP PL), Kathmandu | First edition: 2011 AD; Reprint: 2012 AD; revised ed: 2016 AD; second ed: 2023 AD | TNB | Kumar B. Rai (KBR) | Yes | No first aid mentioned. |
| 8.24 | 8_HPE&CA_23_Eng_Baruwal_Aakar_NA | 8 | HPE&CA*****/ English | APD PL, Ktm | First edition: 2080 BS (2023 AD) Reprint: 2024 | AKB | NA | Yes | No first aid mentioned. |
| 8.25 | 8_HP&CA_24_Eng_Aryal et al_Vidyarthi_NA | 8 | Health, Physical and Creative Arts (HP&CA)*****/ English | Vidyarthi Pustak Bhandar (VPB), Ktm. | First edition: 2023 AD; Second edition: 2024 AD | BA, SND, NPN | Prof. Dr. Ram K. Maharjan (RKM) | Yes | No first aid mentioned. |
| 8.26 | 8_HPE&CA_23_Eng_Kapadi and Maharjan_Green_NA | 8 | Health, Physical Education and Creative Arts (HPE&CA)*****/ English | Green Books (GB), Lalitpur | First edition: 2080 BS (2023 AD) | Bhan Dev Kapadi (BDK), Ram Maharjan (RM) | NA | Yes | No first aid mentioned. |
| 11.27 | 11_HPE_23_Nep_Budhathoki et al_Pinnacle_NA | 11 | Health & Physical Education (HPE)*/ Nepali | Piankal Publication Pvt. Ltd. (PPPL) | 2078\079 BS (2023 AD) | Prof. Dr. Chitra B. Budathoki (CBB), Bishnu P. Wagle (BPW), Dr. Madhav K. Shrestha (MKS) | NA | No | No first aid mentioned. |
| 11.28 | 11_HPE_21_Nep_Kafle&Shrestha_Bhundipuran_NA | 11 | Health & Physical Education (HPE)*****/ Nepali | Bhundipuran Publication (BP) | First edition: 2077 Magh (2021 AD) | Bishnumani Kafle, Mitralal Shrestha | NA | No | No first aid mentioned. |
| 11.29 | 11_HPE_22_Nep_Maharjan et al_Vidhyarthi_NA | 11 | Health & Physical Education (HPE)*****/ Nepali | Vidyarthi Pustak Bhandar (VPB), Ktm. | First edition: 2077 BS (2021 AD); Second edition: 2078 BS (2022 AD) | Prof. Dr. Ramkrishna Maharjan, Somnath Dhakal, Dr. Bhagawan Aryal | NA | No | No first aid mentioned. |
| 11.3 | 11_ATB_24_Eng_Keshari et al_Vidhyarthi_NA | 11 | A Textbook of Biology (ATB)/ English | Vidyarthi Pustak Bhandar (VPB), Ktm. | First edition: 2077 BS (2021 AD); Second edition: 2078 BS (2022 AD); Third edition: 2079 BS (2023 AD); Fourth edition: 2080 BS (2024 AD) | Arvind K. Keshari, Ph.D (AKK), Khaga Raj Ghimire (KRG), Bijay Shankar Mishra (BSM), Kamal K. Adhikari (KKA) | NA | No | No first aid mentioned. |
| 11.31 | 11_HB_21_Eng_Paudell and Tapol_Heritage_NA | 11 | Heritage Biology (HB)/ English | Heritage Publishers and Distributors Pvt. Ltd. (HPD PL), Ktm. | First edition: 2077 BS (2021 AD) | Babu Ram Paudel, PhD, DSc (BRP), Rojan Tapol (RT) | NA | No | No first aid mentioned. |
| 11.32 | 11_PB_21_Eng_Shakya et al._Asmita_NA | 11 | Principles of Biology (PB)/ English | Asmita Books Publisher and Distributors (P) Ltd. (ABPD PL), Ktm. | First edition: 2077 BS (2021 AD) | Madan Shakya, Dr. Kripa R Mehata , Madan Gautam, Keshav R. Pokharel, Keshav Khanal | NA | No | No first aid mentioned. |
| 11.33 | 11_PBB_23_Eng_Gurung and Ghimire_Dreamland_NA | 11 | Pioneer Basic Biology (PBB)/ English | Dreamland Publication Pvt. Ltd. (DP PL), Ktm. | First edition: 2079 BS (2023 AD) | Tek Raj Gurung, Dr Tirtha Raj Ghimire | NA | No | No first aid mentioned. |
| 12.34 | 12_HPE_23_Nep_Budhathoki et al_Pinnacle_NA | 12 | Health & Physical Education (HPE)*****/ Nepali | Piankal Publication Pvt. Ltd. (PPPL) | 2078\079 BS (2023 AD) | CBB, BPW, MKS | NA | Yes | No first aid mentioned. |
| 12.35 | 12_HB_21_Eng_Paudell and Tapol_Heritage_NA | 12 | Heritage Biology (HB)/ English | HPD PL, Ktm. | First edition: 2077 BS (2021 AD); Reprint: 2078 BS (2022 AD) | Babu Ram Paudel, PhD, DSc (BRP), Rojan Tapol (RT) | NA | No | No first aid mentioned. |
| 12.36 | 12_PB_22_Eng_Shakya et al._Asmita_NA | 12 | Principles of Biology (PB)/ English | Asmita Books Publisher and Distributors (P) Ltd. (ABPD PL), Ktm. | First edition: 2078 BS (2022 AD) | Madan Shakya, Dr. Kripa R. Mehata , Madan Gautam, Keshav R. Pokharel, Keshav Khanal | NA | No | No first aid mentioned. |
| 12.37 | 12_ATB_22_Eng_Keshari and Ghimire_Vidhyarthi_NA | 12 | A Textbook of Biology (ATB)/ English | Vidyarthi Pustak Bhandar (VPB), Ktm. | First edition: 2078 BS (2022 AD) | Arvind K. Keshari, (AKK), Khaga R Ghimire (KRG) | NA | No | No first aid mentioned. |
| 12.38 | 12_PB_23_Eng_Keshari and Ghimire_Vidhyarthi_NA | 12 | Practical Biology (PB)/ English | Vidyarthi Pustak Bhandar (VPB), Ktm. | First edition: 2078 BS (2022 AD); Second edition: 2079 BS (2023 AD) | Arvind K. Keshari, (AKK), Jyoti Raj Bhandari (JRB) | NA | No | No first aid mentioned. |
| B.39 | B.Ed.4y_SHPCHS_19_Nep_Budhathoki and Wagle_Pinnacle_87-88 | B. Ed. 4th year | School Health Program and Community Health Survey (SHPCHS)/ Nepali | Piankal Publication Pvt. Ltd. (PPPL) | First edition: 2075 BS (2019 AD) | Prof. Dr. Chitra B. Budathoki (CBB), Bishnu P. Wagle (BPW) | NA | Yes | 87–88 |
| P.40 | Paramed_ETBM-I_22_Eng_Gautam&Bhusal_SPPL_371–372 | Paramedical | Essential Textbook of Basic Medicine-I (ETBM-I)/ English | Samiksha Publication Pvt. Ltd., Kathmandu-29 (SPPL, Ktm.) | First eidition: 2019 AD; Second edition: 2022 AD | Dr. Kapil Gautam, Dr. Aayusma Bhusal | NA | Yes | 371–372 |
| P.41 | Paramed_TFA & BMP_20_Eng_Sapkota and Pathak_VPB_200–201 | Paramedical | A Textbook of First Aid and Basic Medical Procedure (TFA & BMP)/ English | VPB, Ktm. | First edition: 20?? AD; Second edition: 2018 AD; Third edition: 2020 AD | Sochana Sapkota, Sumita Pathak | NA | Yes | 200–201 |
| P.42 | Paramed_TBMP&FA_18_Eng_Pathak_VPB_129–133 | Paramedical | A Textbook of Basic Medical Procedure and First Aid (TBMP & FA)ǂ/ English | VPB, Ktm. | First edition: 2007 AD; Second edition: 2010 AD; Third edition: 2011 AD; Revised edition: 2018 AD | Dr. Tilak Pathak | NA | Yes | 129–133 |
| P.43 | Paramed_ETBMP & FA_19_Eng_Ghimire_SPPL_224–229 | Paramedical | Essential Textbook of Basic Medical Procedure and First Aid (ETBMP & FA)/ English | SPPL, Ktm. | First edition: 2019 AD | Dr. Ishwor Ghimire | NA | Yes | 224–229 |
| P.44 | Paramed_TFN_21_Eng_Mandal&Parsai_SPH_417–419 | Paramedical | A Textbook of Fundamental of Nursing (TFN)**ǂǂ**/ English | Safal Publication House Pvt. Ltd., Ktm. | First edition: 2014 AD; Second edition: 2015 AD; Third edition: 2016 AD; Fourth edition: 2021 AD | Prof. GN Mandal, Prof. Durga Parsai (Subedi) | NA | Yes | 417–419 |
| P.45 | Paramed_TFN_21_Eng_Pathak&Devkota_VPB_524–527 | Paramedical | A Textbook of Fundamentals of Nursing (TFN)**ǂǂ**/ English | VPB, Ktm. | Earlier editions: not given; Revised edition: 2021 AD | Sumita Pathak, Rama Devkota | NA | Yes | 524–527 |
| P.46 | Paramed_TFN_19_Eng_S Pathak_VPB_501–505 | Paramedical | A Textbook of Foundation of Nursing (TFN)**ǂǂǂ**/ English | VPB, Ktm. | First edition: 2018 AD; Second edition: 2019 AD | Sumita Pathak | NA | Yes | 501–505 |
| **Symbols and abbreviations used:** **€**: Given codes representing "Class_Book's name_Last two digits of the year of latest edition of evaluated books_language (**Eng** for English and **Nep** for Nepali)_Author/s_Publisher_pages which contains advice for snakebite prehospital care"; *****: Approved by Curriculum Development Center (**CDC**), Sanothimi, Bhaktapur, Nepal; ******: CDC, Sanothimi, Bhaktapur, Nepal (as an author) produced the book; **ǂ**: For General Medicine (**HA**: Health Assistant), Diploma and Bachelor in Pharmacy, **BPH** (Bachelor in Public Health), **BN** (Bachelor of Nursing), and **PCL and BSc Nursing** (Proficiency Certificate Level (PCL) of Nursing, Bachelor of Science (BSc) in Nursing), **BAMS** (Bachelor of Aayurvedic Medical Science), and **BDS** (Bachelor of Dental Surgery); **ǂǂ**: Based on currently revised curriculum of **CTEVT** (Council for Technical Education and Vocational Training)–PCL Nursing; **ǂǂǂ**: Based on the new curriculum of Bachelor of Science (**BSc**) in Nursing; **NA**: not available or not mentioned or not applicable. | | | | | | | | | |
