# Supplementary figures and images for: Analyzing first aid in textbooks used by non-medical and paramedical students in Nepal: A need of further attention for snakebite management!
Source: PLoS Negl Trop Dis. 2025 Dec 2;19(12):e0013765. doi: 10.1371/journal.pntd.0013765 (PMC12680362; doi:10.1371/journal.pntd.0013765)

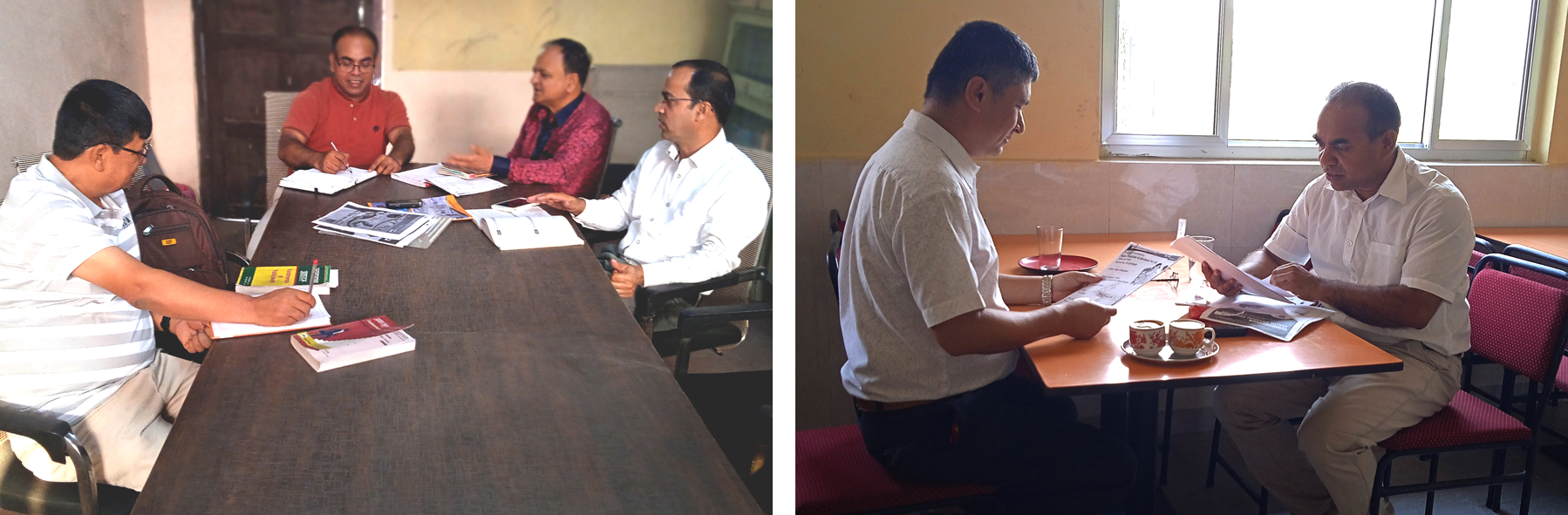

Supplement: S1 Fig — Photograph by DPP. (TIF) [file pntd.0013765.s003.tif]

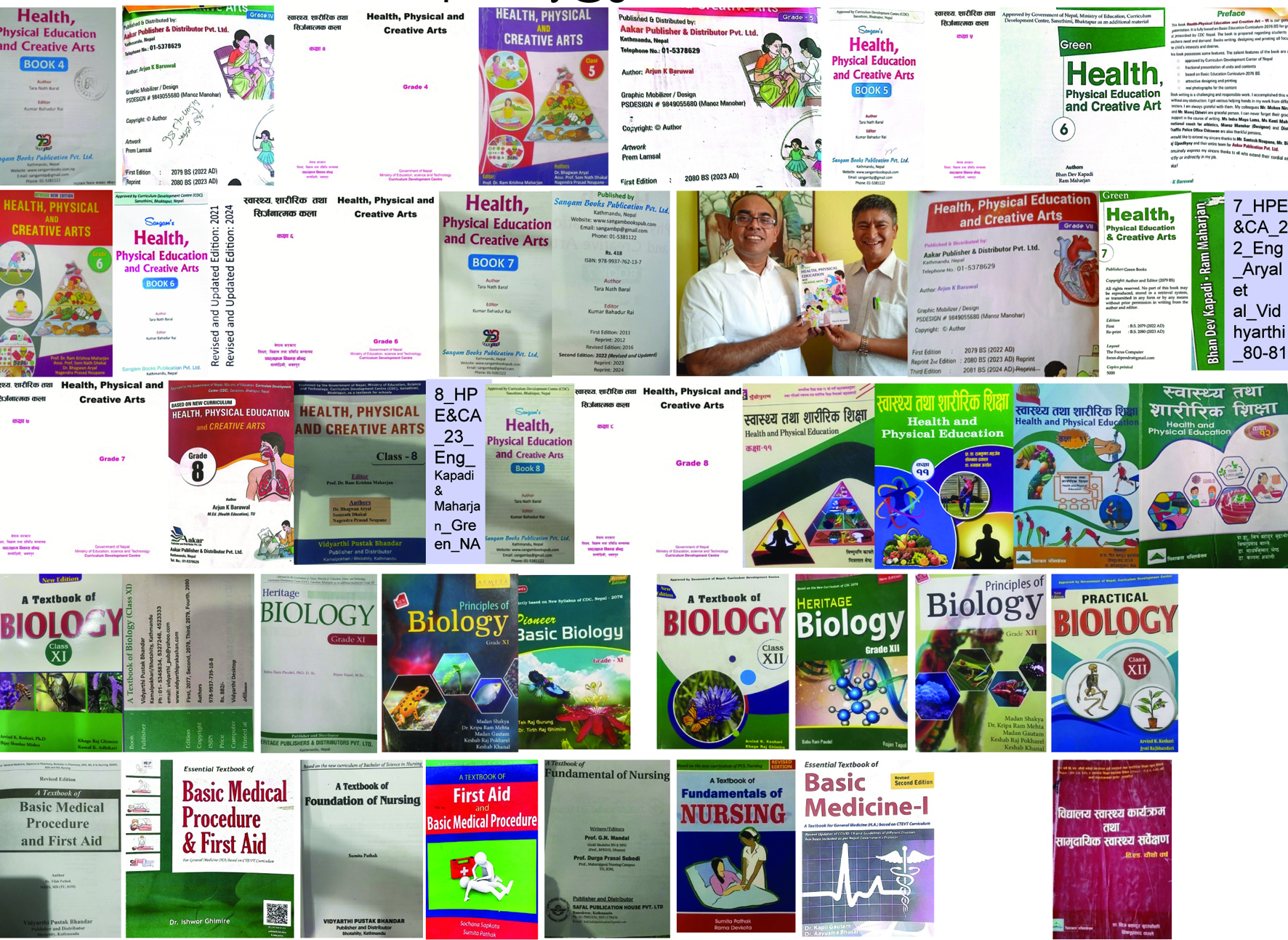

Supplement: S2 Fig — Photograph by DPP. (TIF) [file pntd.0013765.s004.tif]
