# Supplementary material for: Analyzing first aid in textbooks used by non-medical and paramedical students in Nepal: A need of further attention for snakebite management!
Source: PLoS Negl Trop Dis. 2025 Dec 2;19(12):e0013765. doi: 10.1371/journal.pntd.0013765 (PMC12680362; doi:10.1371/journal.pntd.0013765)
Supplement: Attachment — Submitted filename: Revised comments.pdf [file pntd.0013765.s008.pdf]

Title – Analyzing first aid in textbooks used by non-medical and paramedical students in Nepal: a need of further attention for snakebite management!

Manuscript ID- PNTD-D-25-00320R1 for PLOS Neglected Tropical Diseases.

Dear authors,

Thank you. All corrections made by authors.

Dear Editor,

I went through the revised manuscript, and it seems that almost all comments were addressed by the authors.

Thank you.
