# Supplementary material for: Analyzing first aid in textbooks used by non-medical and paramedical students in Nepal: A need of further attention for snakebite management!
Source: PLoS Negl Trop Dis. 2025 Dec 2;19(12):e0013765. doi: 10.1371/journal.pntd.0013765 (PMC12680362; doi:10.1371/journal.pntd.0013765)
Supplement: Attachment — Submitted filename: PNTD-D-25-00320_R0_Author_responses_clean_250710.docx [file pntd.0013765.s007.docx]

# Authors' responses to Reviewers' Comments

## Reviewers' Comments: Reviewer's Responses to Questions

Key Review Criteria Required for Acceptance?

As you describe the new analyses required for acceptance, please consider the following:

## Methods:

-Are the objectives of the study clearly articulated with a clear testable hypothesis stated?

-Is the study design appropriate to address the stated objectives?

-Is the population clearly described and appropriate for the hypothesis being tested?

-Is the sample size sufficient to ensure adequate power to address the hypothesis being tested?

-Were correct statistical analysis used to support conclusions?

-Are there concerns about ethical or regulatory requirements being met?

### Reviewer #1:

need to clearly state how big the data set was, how many pages in each of the 46 textbooks were devoted to snakebite health education.

Line 101: Need to provide definitions of “recommendable advice” and “non recommendable advice”.

Need to inform about the number of textbooks for school students and the number of books for university. Similarly need to inform how many school students were using those books. Such information is necessary to assess/comment on the potential impact at he community level in general.

I suggest adding content analysis rather than a descriptive statistics by quantifying the themes. The dataset is rather small, and the section on first aid is probably a smaller section of a few pages within each book. If descriptive quantitative method is kept, the paper will still benefit from providing some **narrative quotes from within the data**.

#### Authors' responses:

Dear reviewer 1, thank you very much for your critical review comments. We have responded to all of your concerns and advice, respectively, below. Also, we improved the manuscript, too, accordingly. All changes in manuscript are indicated in the track changes.

Pages in each of the 16 out of 46 textbooks devoted to snakebite health education are listed in the 6th column of the "**Table 2:** Scaled commission of inappropriate first aid measures in textbooks containing "Safety and First Aid" unit.". Further, pages devoted to snakebite health education are listed in the 10th column of the "**Supplementary Table 1.** School and university textbooks currently used in Nepal and having potential of advising measures for prehospital care of snakebites.", too.

We provided definitions of “recommendable advice” and “non recommendable advice” in the first paragraph of the introduction (supplemented with the Box 1).

In the second paragraph of the method section, we added the texts describing the number of textbooks for school and university students briefly. Further, we improved the first paragraph of the results, too, by informing the readers about the number of textbooks for school students, paramedical students, and the number of books for university students.

Similarly, in the second paragraph of the method section, we added the texts describing how many school students and paramedical (CTEVT and university) students were using those books. This information is considered to depict the potential impact of this study finding at the school communities primarily.

We considered the suggestion to add content analysis by quantifying the themes in the method section. Accordingly, we improved the second paragraph of **"Hypothesis and data analysis".** Further, in abstract, we replaced "We performed a descriptive analytical review of safety and first aid in textbook. " with " We performed an analytical review of safety and first aid in textbook. ". Similarly, in "Data collection strategies", we replaced "descriptive analytical review of snakebite PHCPs in textbook during ..." with "analytical review of snakebite PHCPs in textbook during ..."

We are pleased to inform reviewer about our listing of narrative quotes within the data in **the Table 1**. We think presentation of quotes and themes (along with the information to track them in the original sources) are adequate to benefit the expected readers of this article.

### Reviewer #2:

The study addresses a pertinent issue regarding the quality of first aid education for snakebite management in Nepal and presents clearly defined objectives. The comparison with a previous study provides valuable context, and the methodological design is appropriate. Nevertheless, the authors are urged to explicitly state the hypothesis in the Introduction, as it is presently only implied.

The authors should elucidate the representativeness of the sample in terms of national coverage and adoption, despite the fact that the sample of 46 textbooks appears to be sufficient for a descriptive analysis.

The Wilcoxon test and two-proportion Z-test with continuity correction are well-executed and suitable statistical analyses; however, the interpretation of the findings would be further strengthened by the inclusion of measures of effect size and the discussion of the **extent of the observed differences**.

Lastly, the investigation comprises interviews with authors and educators, despite the absence of direct research with human or animal subjects. Consequently, the manuscript should specify whether these informants provided verbal or written assent, even if formal ethical approval was not necessary. By addressing these points, the manuscript's scientific rigor and methodological transparency will be improved.

#### Authors' responses:

Dear reviewer 2, thank you very much for your encouraging remarks and critical review comments. We have responded to all of your concerns and advice below, respectively. We improved the manuscript, too, accordingly. All changes in the manuscript are indicated in the track changes.

In line 69–74 (track change version of the manuscript): In the last paragraph in the Introduction, we re-stated the hypothesis.

In the second paragraph of the method section, we described number of school students and paramedical (CTEVT and university) students were using those books. This information depicted the impact of this study at the school and university communities of Nepal. Although national impact, we are not confident that our book selection is 100% nationally representative study. The lacking of the sampling is described in the first bullet of the "Constraints of this study".

We agreed with (and are thankful, too) for his/her suggestion to analyze the effect size which would indicate the practical significance of our research outcome. We improved the last part of data analysis section by stating "Further, we used Cohen’s d to measure the size of the difference between respective proportions of two groups of books. We interpreted the usefulness of these textbooks based on recommended advice without errors and the measure of effect size.". Also, we improved the respective sections of results and the discussion.

Thank you for the suggestions to clarify the ethical concerns associated to interviewed book author and educators, despite the absence of direct research with human or animal subjects in this study. In the ethical approval section of this manuscript, we specified about the informants who provided verbal assent, even if formal ethical approval was not necessary. They were interviewed just to know the recently used textbooks containing safety and first aid advising PHCPs, considering to representing books used in nationwide educational institutions.

### Reviewer #3:

Objectives clearly stated

- Please more clearly describe the 46 textbooks as it pertains to level of education being taught, age groups and under what type of curriculum.

- Supplemental table 1 is very dense and hard to interpret. In the manuscript we need a simply table to describe this data

- Please describe why these districts, schools and Tribhuvan University were chosen. Are these the most populated regions and where the majority of education is provided in Nepal?

How many students are being taught in these regions and how much of the student population in Nepal does it represent?

- I recommend the authors comment on the primary methodology of teaching in these districts and in Nepal. Are most students being taught with physical books? What percentage are being taught via internet or computer resources and does the team know if they are used as online resources for PHCP SBE?

- The sources of guidelines used appear to be Nepal 2019 guidelines and WHO guidelines from 2016.

#### Authors' responses:

Thank you reviewer 3 for your critical review comments. We have responded to all of your concerns and advice below and improved the manuscript, too, accordingly. All changes in the manuscript are indicated in the track changes.

The Supplementary Figure 2 and Supplementary Table 1 clearly illustrate and describe the 46 textbooks to ensure re-visit of each book and concerned pages (when it is needed). In Supplementary Table 1, the level of education being taught is included under the column name as "Classes". These classes obviously represent age groups of the students. However, followed by reviewer, we included the age group in the last part of the "Data collection strategies" of method section in the revised manuscript followed by the formal education system in Nepal. Further, we included the type of curriculum in the second paragraph of the sub-section "Data collection strategies" in method section of the revised manuscript.

Yes, we agreed that the Supplemental table 1 is dense. To ease the interpretation, we already defined the symbols and abbreviations used in the Supplemental table 1. We slightly improved Supplemental table 1 to increase its readability. As suggested by the reviewer, we extracted all key information from the Supplemental table 1 and developed a simple table to describe these data already as "**Table 2:** Scaled commission of inappropriate first aid measures in textbooks containing "Safety and First Aid" unit.".

Aforementioned curricula developed by Curriculum Development Center (CDC) and the Higher Secondary Education Board (HSEB), Bhaktapur, Ministry of Education, Nepal Government, are used nationwide for basic and secondary school education nationwide (elaborated in the second paragraph of the sub-section "Data collection strategies" in method section of the revised manuscript). To cover the types of schools [governmental and non-governmental schools under PABSON, N-PABSAN (The PABSON stands for Private and Boarding School Organization Nepal and N-PABSAN for National Private and Boarding Schools' Association Nepal)], we purposively visited two districts. Same course and curricula are taught in lowly populated Manag and Mustang Districts to highly populated Kathmandu and Bhaktapur Districts including Chitwan and Nawalpur. So, our sampled districts and schools were almost enough to nationwide representation. We described some limitation for the representations in the "Constraints of this Study" section.

Tribhuvan University (TU) is the largest university of Nepal in terms of the number of students' enrollment and nationwide coverage by its institutional and affiliated campuses. Therefore, we visited only the TU to cover the major education sector in higher education of Nepal. There may be similar issues in other university curricula which we could not explore during this study due to time and financial constraints. We highlighted this limitation in the "Constraints of this Study" section so that other similar study can enhance similar studies in future.

The nationwide enrolled students are mentioned in the newly added paragraph (i.e., the second paragraph of the sub-section "Data collection strategies" in method section of the revised manuscript. We believe that this study finding has national impact despite small sample size of districts, schools, and universities.

In Nepal, Information and Communication Technology (ICT) is increasingly recognized as a crucial tool for improving education at all levels, from basic to higher education. In Nepalese schools, internet and computer resources are increasingly being used to support teaching and learning, though access and effective implementation vary across schools and regions. While significant strides have been made in integrating ICT into the education system, challenges remain in terms of infrastructure, teacher training, and equitable access. In these contexts, we found the primary methodology of teaching with physical books in Chitwan, Nawalpur, and all other districts of Nepal. We briefly mentioned it in the last part of second paragraph of the sub-section "Data collection strategies" in method section.

### Reviewer #4:

I understand that the main difference between first aid and pre-hospital care (PHC) lies in the depth and specialization of the care provided. First aid is basic and immediate actions that anyone can perform to stabilize a victim before professional help arrives, while PHC is a more specialized care, performed by trained professionals with advanced equipment and protocols. Therefore, I believe it would be better for the authors to use "first aid" throughout the text.

Could the authors clarify how the 7 people arrived to indicate the evaluated teaching materials? - a health education related book writer, five teachers who used to teach PHCPs for snakebites and other accidental illness in university and schools, and an administrator and science teacher of a school. How can the authors ensure that these chosen people are sufficient to make a satisfactory indication of the materials? Is the number of schools visited sufficient?

#### Authors' responses:

Dear reviewer 4, thank you very much for your remarks and critical review comments. We have responded to all of your concerns and advice below, respectively. And, we improved the manuscript followed by your suggestions and comments. All changes in the manuscript are indicated in the track changes.

We agreed (and understood that) with the main difference between first aid and pre-hospital care (PHC) lies in the depth and specialization of the care provided. As the first aid ***is basic and immediate actions that anyone can perform to stabilize a victim before professional help arrives***, while PHC is a more specialized care, performed by trained professionals with advanced equipment and protocols, we replaced all "PHCPs" with "first aid" throughout the text. Also, we replaced all "prehospital care, pre-hospital care" with the "first aid".

We interviewed seven key informants to get general ideas about the recently used textbooks containing safety and first aid units. These chosen people are sufficient to make a satisfactory indication of the materials because a health education related book writer used to consult/pore over all associated books published in the domain in Nepal, five teachers who used to teach first aid for snakebites and other accidental illness in university and schools are district to national level trainer, too. An administrator and science teacher of a school would know the latest changes and improvement in the school education. So, we believe that these chosen people are sufficient to make a satisfactory indication of the materials.

We did not randomize the school selection. To represent the governmental and non-governmental schools, we purposively visited schools considering time and financial constraints. While considering the representation of schools and universities (as described in the second paragraph of the sub-section "Data collection strategies" in method section of the revised manuscript), the number of schools visited is sufficient to address the issues of the persistent teaching of harmful snakebite first aid in Nepal.

## Results:

-Does the analysis presented match the analysis plan?
-Are the results clearly and completely presented?
-Are the figures (Tables, Images) of sufficient quality for clarity?

### Reviewer #1:

Line 140: Need to inform the reader why quite a few books (11 books that contained no information on safety/first aid and 19 books that did not have snakebite envenoming related information) got identified initially? What was the criteria used to identify.

Line 142: There is a need to inform about the rationale of selecting both school textbooks and university textbooks, highlighting the significance (from community's health education perspective) of grade 6th book relative to the significance of a information included in the curriculum for a bachelor’s degree.

Lines 147-149: Lacks clarity: “for grades 4th, 5th, 8th, 9th, 10th, and B.Ed. 1st–3rd year, recently. Among the currently used textbooks in Nepal, the "Safety and First Aid" unit was included starting the textbooks prescribed for the 4th graders (Supplementary Table 1). But, the advice for snakebite first aid….”

Lines 158-167: some quotes from the reviewed textbooks are needed.

Lines 168-175: comparison with a past study does not add much to the argument. The important is what is the current situation, its reason. If this aspect is to be kept, the previous study needs to be explained in some detail, and the discussion section needs to include some discussion about the factors that led to improvement.

#### Authors' responses:

Dear reviewer 1, thank you very much for your critical review comments. We have responded to all of your concerns and advice, respectively, below. Also, we improved the manuscript, too, accordingly. All changes in manuscript are indicated in the track changes.

We improved the "Line 140 ..." in the result section. To make the readers further clearer, we discussed about books containing no information on safety/first aid and those books that did not have snakebite envenoming related information despite first aid unit was included. These discussions are in the newly added first paragraph of the discussion section. Also, we elaborated the first paragraph of the method section to clarify the criteria used to identify those textbooks.

For addressing "Line 142: There is ...", improved the last segment of the first paragraph of the discussion as "As snakebite is serious, time critical health issues and children are mainly affected widely, the snakebite first aid should be taught beginning one through 12^th^ grades considering their ability of understanding and using the first aid measures. This would help to noticeably decrease the adoption of useless or potentially harmful first aid as they could share about snakebites to their parents, relatives, and neighbor who suffer from snakebites or are at the risk of snakebites. University students are future teachers, too. Hence, the first aid should be taught greater in details with some practical activities to enable them learning/using first aid measures appropriately. All these depict the need of periodic evaluation of curriculum texts by selecting both school and university textbooks, to highlight the significance of first aid education beginning to early childhood to adults."

For clarity, we deleted “for grades 4th, 5th, 8th, 9th, 10th, and B.Ed. 1st–3rd year, recently." from the Result section. This issue is well-described in the first paragraph of the discussion section.

Following your advice, we added some selected quotes from the reviewed textbooks (that are already listed in Table 1, too) in the "Inappropriate first aid of snakebites" of Result section.

Regarding the " comparison with a past study ", Only the "Textbook of Basic Medical Procedure and First Aid p**ublished in** English language edition with the first edition in 2007 was identified book from the list of previous study. So, our comparison is almost independent set of books that were collected between 2010 and 2024. Four books firstly edited in 2011 AD were re-edited time and again and used up to the date of textbooks collection during this study. We opine that comparison between independent set of books could add the argument in some extent. If the previous study needs to be known by the readers in detail, the readers can follow up the open access article easily. However, we have discussed including some factors that led to improvement of text books (in the second paragraph of the discussion).

The current situation of textbooks is well discussed with reasons of revising them for better formal education in Nepal and other snakebite prone countries where status of formal education is similar.

### Reviewer #2:

The results presented are consistent with the stated analysis plan and hypotheses. The authors effectively compare current textbook content with data from a previous study, using appropriate statistical methods to assess differences in the proportions of recommended and non-recommended prehospital care practices. The presentation of the findings is clear, with relevant metrics such as medians, interquartile ranges, and p-values reported. However, while the use of box plots and tables contributes to understanding the data, the quality of the figures should be verified during typesetting to ensure readability and resolution, particularly for Figures 2 and 3, which play a central role in illustrating improvements and persisting deficiencies. Overall, the results are well-structured and support the conclusions drawn, though the visual materials should be reviewed for clarity and publication standards.

#### Authors' responses:

Dear reviewer 2, thank you very much for your critical reviews and remarks.

Following your advice, we cross-checked with resolution and dimension of all figures including figures 2 and 3 (which we already submitted as .tif format). The 300 dpi of each figures (with dimensions for Figure 1 is 15cmx13.8cm, Fig 2 is 15cmx20cm, Fig 3 is 15cmx7cm; Supplementary Fig 1 is 15.25cmx5cm, Supplementary Fig 2 is 15cmx11cm) are under the publishable quality of PLOS NTDs. We will re-improve the quality of these figures during typesetting and ensure readability and resolution by increasing the resolution. If we increased the resolutions at this state, the size of files would be increased beyond the limit for the size of figure files to be uploaded in the PLOS system. When typesetter allows us to upload even the higher size of figure files, we will re-produce these figures within a short period and upload them.

### Reviewer #3:

Please see comments in Conclusions.

#### Authors' responses:

Dear reviewer 3, thank you very much.

### Reviewer #4:

Table 1- The text of the book cited as reference (NATIONAL GUIDELINES FOR SNAKEBITE MANAGEMENT IN NEPAL) by the authors does not show it as recommendable advice, see Page 32 – “Pressure immobilization (PIB) is believed to delay in spread of venom to systemic circulation and PIB method is commonly recommended by many experts in pre-hospital management. However, the pressure-immobilization technique demands special equipment and training and **is not considered practicable for general use in Nepal.** Searching for the material to apply pressure immobilization may cause delay in seeking much needed health care for treatment of envenoming35,36. Moreover, envenoming by cobra and vipers snakes causes local tissue damage and localization of toxin by PIB may worsen tissue damage.”

Na Supplementary Table 1. School and university textbooks currently used in Nepal and having potential of advising measures for prehospital care of snakebites, The books are described and it was possible to identify books from 2010 (15 years ago), are these old books still used? How is the exchange of textbooks carried out in Nepal? Isn't there a book exchange from time to time?

#### Authors' responses:

Dear reviewer 4, thank you very much for your critical review comments. We have responded to both of your concerns below. Also, we improved the manuscript, too, accordingly. All changes in manuscript are indicated in the track changes.

To address the concerns of reviewer 4 about the 32th page of NATIONAL GUIDELINES FOR SNAKEBITE MANAGEMENT IN NEPAL, we removed "the Epidemiology and Disease Control Division (EDCD), Ministry of Health and Population, Nepal Government ... " from method section and we improved the 4th paragraph of the Discussion. We discussed about the controversy of pressure bandaging in support of highly notable reference in the 4th paragraph of the Discussion. Because of the variability of snake venom effects, some recommended first aid practices may be effective for envenoming by some snake species but be harmful for envenoming due to other species. In the context of Nepal, the PIB can be recommended to apply for known bites by kraits (six species inhabit in Nepal) and a coral snake species

because the pressure-immobilisation bandaging (PIB, as a first aid measure) prevents the spread of snake venom (toxins) through the body when the PIB applies enough pressure to compress the lymph vessels in addition to preventing movement of the affected limb. Next, the transportation of snakebite victims to the healthcare facilities that stock and administer antivenom may not be available timely in rural areas (often with poor emergency medical services). These factors have led to the consideration of measures to delay onset of toxicity prior to receiving antivenom therapy in Nepal and other countries with similar situations.

*Since correct application of the PIB delays the envenomation and hence provides enough time to get the patient to appropriate medical assistance owing to deceleration of venom, the use of PIB should be included in the curriculum for the secondary school, paramedical and medical, and university education. The effectiveness of practical-based formal education cannot be compared with informal education performed at community levels.* The recent comprehensive review of articles from 1945 to 2017 (Parker-Cote J and Meggs WJ 2018. First aid and pre-hospital management of venomous snakebites. *Tropical Medicine Infectious Disease*, 3.), it is suggested that the pressure immobilization bandages and related strategies are the best interventions to delay onset of systemic toxicity from venomous snakebites if it is applied appropriately in identified krait and coral snakebite cases (or in all elapid bites causing no necrosis of affected tissues). The Pressure immobilization bandaging (PIB) suggested after animal experimentation by Sutherland et al., in 1979 (Sutherland SK, Coulter AR and Harris RD 1979. Rationalisation of first aid measures for elapid snakebite. Lancet, 313: 183–186.) is also mentioned to be relatively effective in case of elapid snakebites in 2010 and 2016 editions of guidelines published by the World Health Organization [World Health Organization 2010. Guidelines for the prevention and clinical management of snakebites in Africa.

World Health Organization 2016. Guidelines for the management of snakebites. Second ed. New Delhi: World Health Organization, Regional Office for South-East Asia.]. Therefore, our recommendation to application of pressure-immobilisation bandaging (PIB) in identified bites by krait and coral species in Nepal and elsewhere as a pre-hospital care (PHC) to be performed by trained professionals for venomous snakebites, can be applicable in many rural snakebite scenario. From these rural regions, the use of appropriately applied PIB can be useful, too.

Norris et al. [**36.** Norris RL, Ngo J, Nolan K, Hooker G (2005). Physicians and lay people are unable to apply pressure immobilization properly in a simulated snakebite scenario. Wilderness Environ Med 16(1):16–21.]. involved 20 emergency medicine physicians (residents and attendings) and 20 lay volunteers without prior formal medical training and gave a standard printed instructions describing the application of pressure immobilization (PI) for field management of snakebite. The inadequate teaching or training to medical staff or their neglect to care for the neglected snakebite issues might influence the outcome in a study published nearly 20 years ago. Despite the small scale study carried about 20 years ago in certain populations and findings of unsatisfactory performance by the research participants, Norris et al. suggested not to use PI for pitviper bites except for bites inflicted by coral snakes, by sea snakes off the coast of southern California, and by imported exotic elapids not known to cause necrosis. Overall, we would suggest to revise 32 page of guideline suggested by reviewer [NATIONAL GUIDELINES FOR SNAKEBITE MANAGEMENT IN NEPAL, Page 32] for ensuring the optimum level of care to snakebite victims at pre-hospital conditions.

Next, the Canale et al. [**35.** Canale E, Isbister GK, Currie BJ (2009). Investigating pressure bandaging for snakebite in a simulated setting: bandage type, training and the effect of transport. Emerg Med Australas 21(3):184–190.] mentioned that training improved the ability to apply elasticized bandages. Considering these technical difficulties, the PIB should be prescribed for prehospital care by trained professionals (as described in the fourth paragraph of the discussion section).

In the recent studies on two regional hospitals (Pandey DP, Shrestha BR, Acharya KP, et al. 2023. A prospective study of snakebite in a tertiary care hospital in south-western Nepal. *Transactions of the Royal Society of Tropical Medicine and Hygiene*, 117: 435–443. and

Pandey DP, Adhikari B, Pandey P, et al. 2024. Cost of snakebite and its impact on household economy in southern Nepal. *American Journal of Tropical Medicine and Hygiene*, 112: 431–440.), noticeably large number of patients initially accessed to the local healthcare facilities (supplied with no antivenom) where trained healthcare professional can apply the PIB. Next, appropriate formal education using illustrations and simulations (as a part of practical sessions) at secondary schools, paramedical and medical institutions, and universities can enhance the appropriate use of crepe bandage, elasticized bandage or similar local materials for PIB. *Since there is evidence of delaying the envenomation owing to deceleration of venom by correct application of the PIB, the educating needy people formally can provide enough time to carry their patients to the appropriate medical assistance. Therefore, I opine that the mentions of "*Searching for the material to apply pressure immobilization may cause delay in seeking much needed health care for treatment of envenoming35,36." in the guidelines mentioned by the reviewer should be revised.

As snakebite is an emergency situation, the family members or witnesses at rural communities can arrange local materials (or an elastic bandage) while arranging or waiting for the transport. If four-wheel transport was already available, they can use the pressure bandaging within the vehicle. In the recent study on two regional hospitals (Pandey DP, Adhikari B, Pandey P, et al. 2024. Cost of snakebite and its impact on household economy in southern Nepal. *American Journal of Tropical Medicine and Hygiene*, 112: 431–440.), we noticed the multiple hospital attendants of the patients supporting them. So, *PIB should be included in the curriculum for the secondary school, paramedical and medical, and university education.*

Regarding the "Supplementary Table 1", Only a" Textbook of Basic Medical Procedure and First Aid (TBMP & FA) **published in** English language edition with the first edition in 2007 was identified book from the list of previous study (i.e.,15 years ago). Yes, its newer edition versions are still used. Next, a total of 4 books (first edition in 2011 AD) were used for long in their different edition versions. Curriculum Development Center calls authors/editors to provide the draft of books to be approved as a textbook. Those without update or editions are exchanged by those with newly published or newly edited textbooks in Nepal. Of course, there is book exchange from time to time.

## Conclusions:

-Are the conclusions supported by the data presented?
-Are the limitations of analysis clearly described?
-Do the authors discuss how these data can be helpful to advance our understanding of the topic under study?
-Is public health relevance addressed?

### Reviewer #1:

Discussion
Line 180: Lacks clarity: Not clear what is meant by a total of 15 books need immediate re-edition although the advice of harmful, useless or non standard measures in textbooks evaluated during this study were greatly reduced

#### Authors' responses:

Thank you very much for your comments. We have improved to clarify this in the second paragraph of the discussion.

Line 182-185 includes result not discussion

#### Authors' responses:

Thank you very much for your comments. We have moved these texts to the last section of the Result (i.e., subtopic: Comparison of advice for first aid) where we revised the moved texts. The second paragraph of the discussion is clearer now.

Most of the discussion is not aligned to the article’s main focus. The article is not about what is a good or incorrect snakebite first aid advice but about what advice is included in textbooks. Therefore, the discussion needs to be around what might be the reasons for incorrect advice being included in the books, what is the situation in other countries/regions. Similarly, the information about websites the context of the websites appears disjointed from the main argument.

#### Authors' responses:

Thank you very much for your critical comments. We agreed with you that this article is primarily not about what is a good or incorrect snakebite first aid advice but about what advice is included in textbooks. Accordingly, we improved the discussion with the reasons for incorrect advice being included in the books (please, see the last section of the second paragraph of the discussion).

**We could not find relevant literature to know what is the situation in other countries/regions.** While presenting this work in the conferences, the first author of this article (DPP) attempted to know similar ongoing works or published works in the World Congress of International Toxinology being held in Singapore and similar international conference in Madurai, India in 2024. But, supporting information is not known yet. We highlighted this shortcoming in the last of the second paragraph of the discussion and constraints of this study briefly.

Overall, we improved the discussion greatly in this version.

### Reviewer #2:

The conclusions are well supported by the data and highlight the persistence of harmful snakebite first aid advice in textbooks. The authors clearly address the public health relevance and propose meaningful educational interventions. While some limitations are noted, a more explicit discussion of sampling and generalizability would strengthen the interpretation. Overall, the conclusions are valid and relevant to advancing snakebite management education.

#### Authors' responses:

Thank you very much for your critical comments. We discussed about sampling and its representation in the "Data collection strategies" of method section more explicitly. Further, we mentioned about the generalizability at the first bullet of the constraints of this study, considering a need of additional studies covering all available textbooks used nationwide in Nepal. This may encourage other researchers for a grand research project to contribute to education and to reduce the impact of snakebites.

### Reviewer #3:

Conclusions are reasonable and show the need to improve snake envenomation education in these textbooks with updated content. and among these districts.

#### Authors' responses:

Thank you very much for your comments. We further improved the conclusions to make conclusions more reasonable.

I recommend to the authors that they clarify the level of education for these textbooks and they future career pathways for the degrees. For example, "medical undergraduate". Is that a medical student who will then acquire a MD and then go on to being labeled a doctor who will then go to a training program for residency?

Are all these textbooks for primary school level and some college level before graduate school or medical school?

I also think the title needs attention to clarify the level of education for the "textbooks used in Nepal" and also clarify if these are the national textbooks for the entire country. This is an important for the readers to understand immediately when analyzing the findings and also government officials who will undoubtedly need to review your findings.

#### Authors' responses:

Thank you very much for your comments.

In the first and second paragraphs of "Data collection strategies" in the Method section, we clarified the level of education for the textbooks evaluated during this study. Further, we defined the age group of students for the allocated levels. The future career pathways for the Bachelor's Degree students are the Master's Degrees in the respective subjects. The Bachelor's Degree level textbooks included herein does not represent books used by MBBS students. Hence, medical students enrolled in MBBS use books published by foreign writers often. After, MBBS, they will acquire a MD and then go on to a training program for residency, etc. However, early career MBBS students in Nepal are influenced by inadequate knowledge of first aid of snakebite gained during school tenure [reference: Subedi N, Paudel IS, Khadka A, Shrestha U, Mallik VB, et al. (2018) Knowledge of first aid methods and attitude about snake bite among medical students: A cross sectional observational study. Journal of Occupational Medicine and Toxicology 13: 26.].

Following your advice, we improved the title as "*Analyzing first aid in textbooks used by non-medical and paramedical students in Nepal: a need of further attention for snakebite management!*". In the last of the first paragraphs of "Data collection strategies", we clarified about the national textbooks for the entire country. Also, we mentioned it slightly in the last part of the discussion section.

I also recommend a discussion on the snake species which is most commonly encountered from envenomation in Nepal. Speckled cobra, common krait and Russell's viper...

#### Authors' responses:

Thank you very much for your comments. We added some venomous snake species involving in snakebite in Nepal in the fourth paragraph of the Discussion section as:

"... Studies showed that 24–34% of SBE in Nepal involves krait species (*Bungarus caeruleus*, *B. walli*, *B. lividus*, *B. niger*, and *B. fasciatus*) causing nurotoxicity without local necrosis...".

"... Hence, we recommended for using PIB (as pre-hospital care by trained professional) among identified bites by kraits and coral snake (*Sinomicrurus macclellandi*) in Nepal and other countries or regions where these kraits and coral snakes inhabit...."

"... FurtherNext, up to 57% of SBE in Nepal involves cobras (Naja naja, N. kaouthia) [27] which cause cytotoxicity and neurotoxicity. We recommend using LCPI for proven cobra bites as well as all bites by viperid snakesbites (e.g, Daboia russelii, Trimeresurus spp., Ovophis monticola) in Nepal...."

I have concerns about Box 1 and also discussion elements of PIB and LCPI. This is a technique which has shown some efficacy in those bitten by certain Elapids is generally not recommended for Crotalids like the Russell's Viper which is prevalent in Nepal. Author's need to review the Nepal guidelines from 2019 in detail in regards to PIB. " Pressure immobilization (PIB) is believed to delay in spread of venom to systemic circulation and PIB method is commonly recommended by many experts in pre-hospital management. However, the pressure-immobilization technique demands special equipment and training and is **not considered practicable for general use in Nepal.** Searching for the material to apply pressure immobilization may cause delay in seeking much needed health care for treatment of envenoming35,36. Moreover, envenoming by cobra and vipers snakes causes local tissue damage and localization of toxin by PIB may worsen tissue damage"

- in my opinion this is not a clear recommendation on PIB among Elapids in Nepal and references used for PIB and LCPI, 38, 47-49, are not related Nepalese envenomation. Furthermore, looking at the guidelines from Nepal in 2019 it clearly states that considered practical in Nepal.

- I disagree that the authors make recommendations for using PIB and LCPI in Nepal on lines 247 - 250 and throughout manuscript. Is this the purpose of this investigation? Is this something that should be taught and put into curriculum with unclear evidence? The efficacy of PIB and LCPI is not clear and is not a blanket pre-hospital treatment method for all envenomations in Nepal. It may be beneficial in certain circumstances and with Asian Elapids but with Russell's viper there is one paper from Myanmar among n=15. Application standards and techniques are not well defined and trained professionals are needed to understand how much tension to apply and which materials. Tissue necrosis and delay in care have been expressed by other experts in Australasia and authors mention this is their conclusions.

#### Authors' responses:

Thank you very much for your comments. We improved the discussion for PIB and LCPI in the 4th paragraph of the discussion. We suggested PIB and LCPI to personnel trained in PIB and LCPI use. We suggested using *PIB and LCPI as a pre-hospital care by trained professional using special materials to apply an appropriate tension on the bitten limb and bite site, respectively*. Accordingly, we modified elements of PIB and LCPI in Box 1, too.

In the 32th page of NATIONAL GUIDELINES FOR SNAKEBITE MANAGEMENT IN NEPAL, authors mentioned "the pressure-immobilization technique demands special equipment and training and is **not considered practicable for general use in Nepal"** without study of the practicality of this technique for general use **in Nepal even.** We already discussed about the controversy of pressure bandaging in support of highly notable reference in the 4th paragraph of the Discussion. Considering the need of skills and special materials, we recommended to use PIB and LCPI both by trained professionals using appropriate material to apply optimal pressure on the bitten limb or around the bite site. Therefore, we opine to continue pressure-immobilization technique for use by skilled first aider only.

In the last of the fourth paragraph of the discussion, we added the text as:

"Further, we suggest authors of the above mentioned books to provide a note on the need of using PIB and LCPI only after adequate training by general populations vulnerable to snakebites in Nepal. The study on usefulness of these techniques for the general use is needed in Nepal and other countries where their use in general populations are still questionable."

We refrain to decline the suggestion of PIB and LCPI because the pressure-immobilisation bandaging (PIB, as a first aid measure) prevents the spread of snake venom (toxins) through the body when the PIB applies enough pressure to compress the lymph vessels in addition to preventing movement of the affected limb. Next, the transportation of snakebite victims to the healthcare facilities that stock and administer antivenom may not be available timely in rural areas. These factors have led to the consideration of measures to delay onset of toxicity prior to receiving antivenom therapy in Nepal and other countries with similar situations. *Since correct application of the PIB delays the envenomation and hence provides enough time to get the patient to appropriate medical assistance owing to deceleration of venom, the use of PIB should be advised to include (with constraints of requiring special training (skills) and materials) in the curriculum for the secondary school, paramedical and medical, and university education.*

The recent comprehensive review of articles from 1945 to 2017 (Parker-Cote J and Meggs WJ 2018. First aid and pre-hospital management of venomous snakebites. *Tropical Medicine Infectious Disease*, 3.), it is suggested that the pressure immobilization bandages and related strategies are the best interventions to delay onset of systemic toxicity from venomous snakebites if it is applied appropriately in identified krait and coral snakebite cases (or in all elapid bites causing no necrosis of affected tissues). ... Therefore, our recommendation to application of pressure-immobilisation bandaging (PIB) in identified bites by krait and coral species in Nepal and elsewhere as a pre-hospital care (PHC) to be performed by trained professionals for venomous snakebites, can be applicable in many rural snakebite scenario. According to Canale et al. [Canale E, Isbister GK, Currie BJ (2009). Investigating pressure bandaging for snakebite in a simulated setting: bandage type, training and the effect of transport. Emerg Med Australas 21(3):184–190.], training improved the ability to apply elasticized bandages. Therefore, proper teaching of PIB and LCPI use in schools, paramedical institutions, and universities is needed considering the technical difficulties and proved delay in development of toxicity up to 4 hours at least. Therefore, the PIB should be prescribed for prehospital care by trained professionals (as described in the fourth paragraph of the discussion section).

Please, see our detailed responses to similar comments raised by 4th reviewer above.

- I do not see in any of these references that it is recommended to not raise the extremity above the heart. This may be a practice that is reasonable, but also do we have evidence to state we should not do something.

#### Authors' responses:

Thank you very much for your comments. We discussed the issue of "raising extremity above the heart" in the 6th paragraph of the discussion. We found that Parker-Cote and Meggs (2018) [Parker-Cote J and Meggs WJ 2018. First aid and pre-hospital management of venomous snakebites. *Tropical Medicine Infectious Disease*, 3.] made a conclusion after a comprehensive review of relevant sources to position the extremity below or at the level of the heart according to the individual situations. For snakebites with severe and potentially fatal systemic toxicity, the systemic toxicity might be delayed by positioning the extremity below the heart, whereas for snakebites with severe local tissue damage and less systemic toxicity, positioning the extremity below the heart could increase local toxicity. For the severe local tissue damage and less systemic toxicity, the positioning of extremity at the level of heart can minimize the tissue damage. We improved the 6th paragraph of the discussion.

- It is evident that the authors have put a lot of work into reviewing these textbooks but

#### Authors' responses:

Thank you very much for your note.

### Reviewer #4:

In conclusion the authors wrote “The persistent teaching of harmful interventions for prehospital care of snakebites in schools and universities of Nepal suggests a neglect of education system and barriers to prehospital care of snakebites.” I believe that describing negligence in the educational system is very strong and they should review their conclusions.

#### Authors' responses:

Dear reviewer 4, thank you very much for your remarks on the conclusions. We also believe that our highlight of negligence in the educating people will be read and used by education and health policy makers in Nepal and contribute to improving teaching and educational materials used in this country. This study can be an example to improve other similar educational materials in Nepal and other countries where education is inadequately supervised/poorly monitored.

# Editorial and Data Presentation Modifications?

Use this section for editorial suggestions as well as relatively minor modifications of existing data that would enhance clarity. If the only modifications needed are minor and/or editorial, you may wish to recommend “Minor Revision” or “Accept”.

## Reviewer #1:

I suggest adding content analysis rather than a descriptive statistics by quantifying the themes only. The dataset is rather small, and the section on first aid is probably a smaller section of a few pages within each book. If descriptive quantitative method is kept, the paper will still benefit from providing some narrative quotes from within the data.

Lines 158-167: some quotes from the reviewed textbooks are needed.

Lines 168-175: comparison with a past study (which is not explained to any details) is least useful and does not add much to the argument.

#### Authors' responses:

Thank you very much for your comments. We removed descriptive from the first line of the "Data collection strategies". As we quantified the theme, the thematic approach of analysis would be better than content analysis. Following your advice, we added some selected quotes from the reviewed textbooks (that are already listed in Table 1, too).

Regarding the " comparison with a past study ", Only the "Textbook of Basic Medical Procedure and First Aid p**ublished in** English language edition with the first edition in 2007 was identified book from the list of previous study. So, our comparison is almost independent set of books that were collected between 2010 and 2024. We consider that comparison between independent set of books have indicated improvement of text books, but requiring editions in 15 books at least. If we did not compare, it would not give the pathways of improvement in educational tools used in Nepal. So, we think that comparing gives some ideas and arguments to its readers.

## Reviewer #2:

The manuscript is generally well written and clearly organized. However, I suggest a careful language revision to improve clarity and reduce redundancy in some sections, particularly the Introduction and Discussion. Some grammatical inconsistencies and overly long sentences may hinder reader comprehension.

#### Authors' responses:

Thank you very much for your notes and comments on the language use. We extensively revised this manuscript with special focus on its English usage and spelling errors in several occasions. All improvements are indicated in the track changes.

Figures and tables are informative, but I recommend ensuring high-resolution versions are provided, especially for Figures 2 and 3, which are central to the study's message. Table captions could also be slightly expanded to ensure clarity without reference to the main text.

Additionally, the “Box 1” summary is a valuable tool for readers and should be carefully formatted for visual clarity. With these minor editorial adjustments, the manuscript will be suitable for publication.

#### Authors' responses:

Thank you very much for your comments. We uploaded all figures to the Preflight Analysis and Conversion Engine (PACE) digital diagnostic tool, <https://pacev2.apexcovantage.com/>. The PACE ensured that figures meet PLOS requirements.

We developed "Box 1" in the .tiff format to make it printed at higher quality. When typesetter further comments on unreadable texts in digital and print versions, we will re-improve the Box 1. In this version, we removed the references contained in the Box 1 because the respective references are mentioned in the discussion section.

## Reviewer #3:

(No Response)

#### Authors' responses:

Thank you.

## Reviewer #4:

 Minor Revison

#### Authors' responses:

Thank you.

# Summary and General Comments

Use this section to provide overall comments, discuss strengths/weaknesses of the study, novelty, significance, general execution and scholarship. You may also include additional comments for the author, including concerns about dual publication, research ethics, or publication ethics. If requesting major revision, please articulate the new experiments that are needed.

## Reviewer #1:

English editing is required

#### Authors' responses:

Thank you very much for your comments on the English language use. We concentrated to improve it. We hope that the revised version contain more formal and error free English use.

## Reviewer #2:

The study addresses a relevant and underexplored public health issue with appropriate methodology and sound analysis. The results support the conclusions, and public health implications are clearly stated. Minor revisions are needed to improve hypothesis clarity, discuss sampling limitations more explicitly, enhance language precision, and ensure figure quality. No ethical concerns identified.

#### Authors' responses:

Thank you very much for your comments. We performed minor revisions to improve hypothesis clarity in the last of the last paragraph of introduction section, discussed sampling limitations more explicitly, and enhanced language precision.

Thank you very much for your comments. We uploaded all figures to the Preflight Analysis and Conversion Engine (PACE) digital diagnostic tool, <https://pacev2.apexcovantage.com/>. The PACE ensured that figures meet PLOS requirements.

## Reviewer #3:

I commend the authors for their work as it is valuable that our school age children and those training in premedical and paramedical fields know evidence-based pre-hospital practices for snake bite victims.

I have concerns with the methods and also with the recommendations for PIB and LCPI. In my opinion, these techniques have not been well studied among Nepalese snake bites and have shown limited evidence of their efficacy. There are real concerns for those without training on appropriate techniques for the placement of PIB and LCPI that they will lead to tissue necrosis and delay in care. It is also unclear why the authors are recommending it's use as the 2019 Nepalese guidelines are not clear about recommending this practice and frankly say it is "not practical for general use in Nepal". My thoughts align with this recommendation that it is not for general use, needs specialized training and likely will only benefit certain snake bite victims. Unfortunately, I have to recommendation rejection at this time.

#### Authors' responses:

Thank you very much for your commendation.

We have improved the methods greatly. I think it is more readable and increases repeatability of this study, too.

So far as the concern of Reviewer 3 with the recommendations for PIB and LCPI, we would recommend these typical measures for special conditions.

Using pressure-immobilization bandaging (PIB) for first aid can delay the spreading of venom injected by neurotoixic snakes **for almost six hours** [Sutherland SK, Coulter AR and Harris RD 1979. Rationalisation of first aid measures for elapid snakebite. Lancet, 313: 183–186.

Murrell G 1981. The effectiveness of the pressure-immobilization first-aid technique in the case of a Tiger Snake bite. *Medical Journal of Australia*, 2: 295–295.].

[Struan Sutherland (1936-2002) was the doyen of medical research in the field of envenomation and the ultimate authority on the medical management of envenomated victims in Australia for almost 3 decades. Dr. Sutherland and his colleagues invented the pressure-immobilisation technique of first-aid for snakebite. This creative, simple but safe and effective technique revolutionised first-aid management of snakebite because the PIB, as a first aid measure, prevents the spread of snake venom (toxins) through the body because the properly used PIB applies enough pressure to compress the lymph vessels in addition to preventing movement of the affected limb. This measure is widely recommended in the guidelines of WHO which are prepared after through discussions of related scientists and medical doctors experienced with managing snakebite in South Asian contexts, too. Please, find the reference details as:

[World Health Organization 2010. Guidelines for the prevention and clinical management of snakebites in Africa.

World Health Organization 2016. Guidelines for the management of snakebites. Second ed. New Delhi: World Health Organization, Regional Office for South-East Asia.]

Species composition and their venom nature is almost similar in South Asian region. Nepal is not unique at all if we talk about the effect of snake venom in general. Next, the delay of spreading venom injected by certain neurotoixic snake species [in Nepalese context, kraits (Bungarus species) and coral snake (Sinomicrurus species) bites] **for up to six hours is highly notable** for events taking place in the rural areas where people should wait for the transportation for long and takes several hours to bring snakebite victims to the healthcare facilities that stock and administer antivenom. Considering these factors, we have led to consider prescribing PIB (and LCPI) to be used by trained personnel using special equipment [i.e., crepe bandage or any similar elasticized local materials (in case of household or nearby local dispensary without crepe bandage); locally available materials for sling or splint] in order to delay onset of toxicity prior to receiving antivenom therapy in Nepal and other countries with similar circumstances.

Since PIB and LCPI needs more skills it should be taught serially from early classes to the university levels with its pros and cons for using or not using it properly. This can prepare large number of people able to apply it in the community within certain years of teaching it formally. Only ad hoc awareness campaign with limited visual aids may not be enough to use these first aid measures widely accepted in the scientific communities.

From "Data coding and their clustering and interpretations " of method section, we removed "... and the EDCD [9]" The 9th reference was the Nepalese guidelines published in 2019 by a governmental body which we found not clear about recommending PIB and LCPI. But, like many global experts in the field, we are convinced to recommend PIB and LCPI to use for specific snakebite and to apply by trained professional using appropriate materials. Because of live saving for affected victims at remote areas with inadequate transport facilities, these techniques should be transferred to students of all levels by teaching them practically and formally to enable the general people inhabiting snakebite prone regions of Nepal and similarly affected remote areas elsewhere for applying these first aid measures until more effective measures are invented and approved by formal scientific bodies like the World Health Organization, International Society of Toxinology, etc.

## Reviewer #4:

The topic addressed is interesting and pertinent. Initial care in snakebite accidents is rarely addressed and the use of educational materials seems to be a support point to prepare young people for this emergency care. Some details and adjustments are necessary to better understand the research as listed in the other items of the report.

#### Authors' responses:

Thank you very much for your comments. We improved the manuscript noticeably. We hope that it is more satisfying than the previous version.

# PLOS authors have the option to publish the peer review history of their article ([what does this mean?](https://track.editorialmanager.com/CL0/https:%2F%2Fjournals.plos.org%2Fplosntds%2Fs%2Feditorial-and-peer-review-process%23loc-peer-review-history/1/010f019759185698-11469e33-3c8f-4dd0-a222-d0c014e981ad-000000/m_odzWxqo7Wck1B8EZFqznrrv010hkm2BsBcsEK5tes=214)). If published, this will include your full peer review and any attached files.

If you choose “no”, your identity will remain anonymous but your review may still be made public.

**Do you want your identity to be public for this peer review?** For information about this choice, including consent withdrawal, please see our [Privacy Policy](https://track.editorialmanager.com/CL0/https:%2F%2Fwww.plos.org%2Fprivacy-policy/1/010f019759185698-11469e33-3c8f-4dd0-a222-d0c014e981ad-000000/G9SrjFEIebRudp2VRD-645B1WTezAhuyPxtIOCXPpdA=214).

## Reviewer #1: No

## Reviewer #2: No

## Reviewer #3: No

## Reviewer #4: No

[NOTE: If reviewer comments were submitted as an attachment file, they will be attached to this email and accessible via the submission site. Please log into your account, locate the manuscript record, and check for the action link "View Attachments". If this link does not appear, there are no attachment files.]

#### Authors' responses:

Thank you.

# Figure resubmission:

While revising your submission, please upload your figure files to the Preflight Analysis and Conversion Engine (PACE) digital diagnostic tool, <https://pacev2.apexcovantage.com/>. PACE helps ensure that figures meet PLOS requirements. To use PACE, you must first register as a user. Registration is free. Then, login and navigate to the UPLOAD tab, where you will find detailed instructions on how to use the tool. If you encounter any issues or have any questions when using PACE, please email PLOS at [figures@plos.org](mailto:figures@plos.org). Please note that Supporting Information files do not need this step. If there are other versions of figure files still present in your submission file inventory at resubmission, please replace them with the PACE-processed versions.

#### Authors' responses:

Thank you very much for your comments. We uploaded all figures to the Preflight Analysis and Conversion Engine (PACE) digital diagnostic tool, <https://pacev2.apexcovantage.com/>. The PACE ensured that figures meet PLOS requirements.

# Reproducibility:

To enhance the reproducibility of your results, we recommend that authors of applicable studies deposit laboratory protocols in [protocols.io](http://protocols.io/), where a protocol can be assigned its own identifier (DOI) such that it can be cited independently in the future. Additionally, PLOS ONE offers an option to publish peer-reviewed clinical study protocols. Read more information on sharing protocols at <https://plos.org/protocols?utm_medium=editorial-email&utm_source=authorletters&utm_campaign=protocols>

*In compliance with data protection regulations, you may request that we remove your personal registration details at any time.* [*(Remove my information/details)*](https://track.editorialmanager.com/CL0/https:%2F%2Fwww.editorialmanager.com%2Fpntd%2Flogin.asp%3Fa=r/1/010f019759185698-11469e33-3c8f-4dd0-a222-d0c014e981ad-000000/uwYbfzyUBXLRDjvoH-SjDWpjbOU1RRtMGM11WvQ7DQs=214)*. Please contact the publication office if you have any questions.*

#### Authors' responses:

Thank you. We have no laboratory protocols to be assigned. Supplementary Tables, Supplementary Figures, Tables, and Figures displays major information. The rest of the information can be accessed by contacting the corresponding authors, which is mentioned in the "Data availability" section.
