# Supplementary material for: Analyzing first aid in textbooks used by non-medical and paramedical students in Nepal: A need of further attention for snakebite management!
Source: PLoS Negl Trop Dis. 2025 Dec 2;19(12):e0013765. doi: 10.1371/journal.pntd.0013765 (PMC12680362; doi:10.1371/journal.pntd.0013765)
Supplement: Attachment — Submitted filename: PNTD-D-25-00320R1_Response_to_reviewers_250820_.docx [file pntd.0013765.s009.docx]

# Author responses to reviewer's Responses to Questions

## Key Review Criteria Required for Acceptance?

As you describe the new analyses required for acceptance, please consider the following:

## Methods

-Are the objectives of the study clearly articulated with a clear testable hypothesis stated?
-Is the study design appropriate to address the stated objectives?
-Is the population clearly described and appropriate for the hypothesis being tested?
-Is the sample size sufficient to ensure adequate power to address the hypothesis being tested?
-Were correct statistical analysis used to support conclusions?
-Are there concerns about ethical or regulatory requirements being met?

### Reviewer #1:

Method is described well.

### Authors' responses:

Thank you very much reviewer #1.

### Reviewer #3:

Authors have provided clarifications for Methods as requested by peer reviewers. I find the changes made to be sufficient. No additional comments

### Authors' responses:

Thank you very much reviewer #3.

### Reviewer #5:

yes, addressed reviewer comments.

### Authors' responses:

Thank you very much reviewer #5.

## Results

-Does the analysis presented match the analysis plan?
-Are the results clearly and completely presented?
-Are the figures (Tables, Images) of sufficient quality for clarity?

### Reviewer #1:

At the first review, it was suggested that a distinction be made between what ought to be included in a textbook for 4th/6th/7th grade students compared to the information that ought to be included in a text book for a bachelor degree about health. For example, information about AV, Antivenom availability at a health centre, how to transfer a patient to the facility may not be needed for a 4th or 7th grade student. Hence, evaluation of textbook may only be with reference to what ought to be / is appropriate for a particular level of schooling. For the same reason, judging a textbook whether it has complete or partial information needs to be contextualised with reference to what is the appropriate level of information for that grade. Results still lack clarity in this regard.

### Authors' responses:

Thank you very much reviewer #1 for critical reviews and constructive suggestions. Following your creative suggestions, we improved the initial paragraphs of the discussion and added a paragraph at the end of the Result section with a subtopic entitled "First aid measures appropriate for a particular level of formal education". We highlighted the need of distinction between first aid measures to be included in a textbook for non-medical (basic level and secondary level and university students) and paramedical students.

Basic level education includes grade 1^st^ through 8^th^ grade students. The 1st through 5th graders are known as primary level students and 6th to 8th graders are called lower secondary level students. And, the 9th through 12th graders are known as secondary level students. Worldwide and in Nepal, children are the most vulnerable and affected population due to snakebites. So, we suggested to be focused on teaching prevention of snakebite in general to primary level students and gradually more advance courses on first aid and prevention measures aimed to higher level students referring to Box 1 and published sources.

### Reviewer #3:

Authors have made several changes and added material for the Results sections as indicated by the peer reviewers and academic editor. Some limitations are discussed which are acknowledged by the author team. Results of the study will shine light on T. cruzi infection and variability within this region of Brazil. Mixed infection among the cohort is interesting. The diversity of DTUs within this region is also important.

### Authors' responses:

Thank you reviewer #3 for the green highlighted comments. Your yellow-highlighted comments are irrelevant to our study. Probably, this is mistakenly copy and paste of comments for similar review of T. cruzi infection in Brazil.

### Reviewer #5:

Corrected accordingly.

### Authors' responses:

Thank you very much reviewer #5.

## Conclusions

-Are the conclusions supported by the data presented?
-Are the limitations of analysis clearly described?
-Do the authors discuss how these data can be helpful to advance our understanding of the topic under study?
-Is public health relevance addressed?

### Reviewer #1:

Discussion section is rather too long. The research was not about what is a correct or incorrect method of first aid. Hence, all the detailed write up about the accurate/useful/harmful method of first aid could be removed from the discussion section. The discussion needs to **focus on why/why not textbook contain/not contain health related information and why incorrec**t information gets included, what are the ways to manage to that etc. Additionally, results are repeated in the discussion. Such repeat description of results needs to be removed.

### Authors' responses:

Thank you very much reviewer #1.

We removed the repeated description of results and noticeably shortened the discussion section in this version of the manuscript.

In the initial paragraphs of discussion, we have focused on why/why not textbook contain/not contain first aid related information and why incorrect information gets included in those textbooks. Also, we highlighted the ways to address these lacking.

We agreed with the reviewer #1 that this research was not about what is a correct or incorrect method of first aid. However, to convince the editors or authors of book and education policy makers, all detailed write up about the accurate/useful/harmful method of first aid including some first aid approaches requiring intensive teaching would be an important component of this manuscript. So, we did not remove these discussions. In resource poor nation like Nepal, this article is expected to be a strongly persuading educational tool to bring changes in formal education of snakebite nationwide and contribute to the WHO's goal of reducing snakebite related morbidity and mortality by half by 2030.

### Reviewer #3:

Conclusions are reasonable. Limitations are also mentioned.

One correction that needs to be made is the citation found on line 341. it is the wrong reference to T. melanica. Should be: "Valença-Barbosa C, Finamore-Araujo P, Moreira OC, Alvarez MVN, Borges-Veloso A, Barbosa SE, Diotaiuti L, de Souza RCM. High Parasitic Loads Quantified in Sylvatic Triatoma melanica, a Chagas Disease Vector. Pathogens. 2022 Dec 8;11(12):1498. doi: 10.3390/pathogens11121498. PMID: 36558833"

### Authors' responses:

Thank you reviewer #3 for the green highlighted comments. Your yellow-highlighted comments are irrelevant to our study. Probably, this is mistakenly copy and paste of comments for similar review of other article.

### Reviewer #5:

yes.

### Authors' responses:

Thank you very much reviewer #5.

# Editorial and Data Presentation Modifications?

Use this section for editorial suggestions as well as relatively minor modifications of existing data that would enhance clarity. If the only modifications needed are minor and/or editorial, you may wish to recommend “Minor Revision” or “Accept”.

### Reviewer #1:

See the comments about the result section i.e how to [analyze] present the results with reference to grade/level of education

### Authors' responses:

Thank you very much reviewer #1.

### Reviewer #3:

Accept

### Authors' responses:

Thank you very much reviewer #3.

### Reviewer #5:

yes.

### Authors' responses:

Thank you very much reviewer #5.

# Summary and General Comments

Use this section to provide overall comments, discuss strengths/weaknesses of the study, novelty, significance, general execution and scholarship. You may also include additional comments for the author, including concerns about dual publication, research ethics, or publication ethics. If requesting major revision, please articulate the new experiments that are needed.

### Reviewer #1:

Overall, the comments and issues raised at the first review are addressed well.

### Authors' responses:

Thank you very much reviewer #1.

### Reviewer #3:

Authors have sufficiently addressed all the suggestions and clarifications provided by the peer reviewers and academic editor. Study has validity and will add to the medical literature for this region of Brazil and the complexity of T. cruzi infection in humans.

### Authors' responses:

Thank you reviewer #3 for the green highlighted comments. Your yellow-highlighted comment may be irrelevant if you connected it with T. cruzi. Probably, you mistakenly copied comments for similar review of T. cruzi herein while you worked for two tasks of peer-reviewing together.

### Reviewer #5:

yes.

### Authors' responses:

Thank you very much reviewer #5.

# Publication of the peer review history

PLOS authors have the option to publish the peer review history of their article ([what does this mean?](https://track.editorialmanager.com/CL0/https:%2F%2Fjournals.plos.org%2Fplosntds%2Fs%2Feditorial-and-peer-review-process%23loc-peer-review-history/1/010f01987b8e85bc-70762156-10de-4e55-b472-7cf08509f2a1-000000/guTPp5QYYnLTMxsY9uwE-RTzBKpgNc6M1-jWryo0PYQ=222)). If published, this will include your full peer review and any attached files.

If you choose “no”, your identity will remain anonymous but your review may still be made public.

**Do you want your identity to be public for this peer review?** For information about this choice, including consent withdrawal, please see our [Privacy Policy](https://track.editorialmanager.com/CL0/https:%2F%2Fwww.plos.org%2Fprivacy-policy/1/010f01987b8e85bc-70762156-10de-4e55-b472-7cf08509f2a1-000000/GBJDPb0NQIjWR_JQtDbUL-RgFDULZGmi3B9zwHTuxEA=222).

### Reviewer #1:

No

### Authors' responses:

Thank you reviewer #1.

### Reviewer #3:

**Yes:** Norman L. Beatty, University of Florida College of Medicine

### Authors' responses:

Thank you reviewer #3.

### Reviewer #5:

No

### Authors' responses:

Thank you reviewer #5.

## [NOTE:

If reviewer comments were submitted as an attachment file, they will be attached to this email and accessible via the submission site. Please log into your account, locate the manuscript record, and check for the action link "View Attachments". If this link does not appear, there are no attachment files.]

## Reviewer #5 comments in an attachment file:

Dear authors,

Appreciated your efforts in doing such an analysis on a very uncommon task.

My concerns are:

1. Abstract/methodology- better to write p values this way- p<0.001, without colon. Need to consider other places in the paper as well (mentioned in different ways in different places)
2. Introduction- The Global picture was there. What are the findings in Nepal, and which snakes caused them?

### Authors' responses:

Thank you very much reviewer #5.

We improved writing p values. We mentioned it as p<0.001 for p values smaller than 0.001 and p=0.001 for p values exactly same as 0.001, without colon. We considered it placed it in all sections of the paper. Now, p values are mentioned in same way in the entire manuscript.

As suggested by the reviewer #5, we improved line 45 to 54 section of the Introduction, some findings in Nepal and common medically relevant snakes causing envenomation in Nepal.
