# Supplementary material for: Analyzing first aid in textbooks used by non-medical and paramedical students in Nepal: A need of further attention for snakebite management!
Source: PLoS Negl Trop Dis. 2025 Dec 2;19(12):e0013765. doi: 10.1371/journal.pntd.0013765 (PMC12680362; doi:10.1371/journal.pntd.0013765)
Supplement: Attachment — Submitted filename: PNTD-D-25-00320R2_Response to Reviewers_251007.docx [file pntd.0013765.s010.docx]

# Author Responses Reviewers' comments:

# A] Key Review Criteria Required for Acceptance?

As you describe the new analyses required for acceptance, please consider the following:

## Methods

-Are the objectives of the study clearly articulated with a clear testable hypothesis stated?
-Is the study design appropriate to address the stated objectives?
-Is the population clearly described and appropriate for the hypothesis being tested?
-Is the sample size sufficient to ensure adequate power to address the hypothesis being tested?
-Were correct statistical analysis used to support conclusions?
-Are there concerns about ethical or regulatory requirements being met?

### Reviewer #1:

Overall, good description of methods
The information about schools and universities provided in the method section does not belong here. It could be placed at the end of the introduction section as part of the 'rationale' [potential significance of this research]

#### Author responses:

Thank you so much for the intensive review and creative suggestions. We adjusted the information about schools and universities provided in the method section in result and introduction sections.

We removed "The 38 out of 46 books were used by students enrolled for school curricula, seven books by paramedical students enrolled for the CTEVT curricula and university curricula, and one book used by students enrolled for Tribhuvan University curricula (Supplementary Tables 1–2)." from the second paragraph of the method section. And, we adjusted this section in the first paragraph of the Result section.

Also, we removed " Basic (grades 1–8) and secondary (grades 9–12) education system aims formal education in Nepal through publicly and privately funded schools and religious schools. In the academic year 2024/25, a total of 35,447 schools [community and religious schools: 27,298 (77.0%), institutional (aka private) schools: 8,149 (23.0%); cited in: https://edusanjal.com/blog/education-levels-nepal/; accessed on: 18th June 2025] enrolled 7,091,000 students nationwide (cited in: https://www.nrb.org.np/contents/uploads/2022/07/Education.xlsx; accessed on: 18th June 2025]. The total CTEVT students enrolled in Diploma/PCL (Proficiency Certificate Level) in Health/Nursing (i.e., Diploma in Pharmacy/PCL of Pharmacy, PCL of Nursing, Community Health Worker, Community Medicine Assistant, Health Assistant, Aayurvedic Health Assistant, Aayurvedic Auxillary Health Worker] was 12,747 (cited in: Appendix 4, available at: https://ctevt.org.np/documents/ctevt-annual-report-2081). Next, the number of students enrolled yearly for curriculum of Bachelor's Degree in Health/Nursing [Bachelor of Pharmacy (B. Pharm) and Bachelor of Science in Nursing (BSc Nursing), Bachelor of Nursing Science (BNS), Bachelor of Public Health (BPH), Bachelor of Ayurvedic Medicine and Surgery (BAMS)] in different Nepalese universities was 3,041 (Supplementary Table 2). Nationwide, 500 institutions affiliated to TU currently offer Bachelor of Education (B.Ed.) in Nepal (https://edusanjal.com/course/bachelor-of-education-bed-tribhuvan-university/). There was primary methodology of teaching with physical books in Nepalese educational institutions although Information and Communication Technology (ICT) is increasingly recognized as a crucial tool for improving education at all levels, from basic to higher education in this country." from the second paragraph of the method section and shifted it to the second-last paragraph of the introduction section.

### Reviewer #5:

Given comments addressed.

#### Author responses:

Thank you so much for the next intensive review and approval of method section.

## Results

-Does the analysis presented match the analysis plan?
-Are the results clearly and completely presented?
-Are the figures (Tables, Images) of sufficient quality for clarity?

### Reviewer #1:

Results and analysis align with the objective of the research.

#### Author responses:

Thank you so much for the next intensive review and approval of this section.

### Reviewer #5:

Given comments addressed.

#### Author responses:

Thank you so much for the next intensive review and approval of this section.

## Conclusions

-Are the conclusions supported by the data presented?
-Are the limitations of analysis clearly described?
-Do the authors discuss how these data can be helpful to advance our understanding of the topic under study?
-Is public health relevance addressed?

### Reviewer #1:

The first paragraph of the discussion is rather a 'recommendation'. This needs to be moved towards the end of the discussion section.
A recommendation states that first aid education should be included in the books for the first grade. This recommendation needs to be based on evidence (reference) of effectiveness of public health prevention / first aid methods for snakebite or other similar public health issues for children at first grade level.
Discussion still includes lengthy description of first aid methods. That description is not relevant and is outside the scope of this work. The reader could be referred to WHO guidelines to learn about and include in their books the correct methods. Instead, the discussion section should include a para or two about the possible reasons to authors including incorrect information or inadequate information in their books.

#### Author responses:

Thank you so much for the next intensive review and creative suggestions to improve the Discussion. We significantly re-improved it following your advice.

We agreed that the first two paragraphs of the discussion are discussions with 'recommendations'. Agreeing with your advice, we moved these paragraphs at the end of the discussion section.

We consider that a recommendation of prevention and first aid education to be included in the books for the first grade because early awareness of these children can discourage to play in bushy areas, enhance habit of reporting animal bites to their parents or teacher (PI of this article has witnessed no report of snakebite while playing outdoors to their parents resulting in their death). Therefore, we wished to recommend basic and simple measures of prevention and first aid (requiring no special skills, training, and supervision to apply them) based on evidence of ability to learn these basic first aid and prevention measures by the kindergarten children aged 4-5 years [1]. Next, snakebite envenoming is a serious, time critical health issue affecting mainly children [2,3]. Therefore, learning of snakebite prevention and first aid in schools would start from the grade 1. But, in Nepal, it mainly start from 7^th^ grade (expect two English edition "Health, Physical, and Creative Arts" targeted to 6^th^ grader contained snakebite first aid measures; Table 2, Supplementary Table 1). Hence, the snakebite first aid and prevention measures should be taught beginning 1^st^ grade through 12^th^ grade (non-medical) students considering their ability of understanding of those measures according to their mental growth by ages and needs. The slightly improved discussion in this context is mentioned in the second last paragraph of the discussion section.

In the second paragraph of the discussion section, we discussed about the possible reasons to authors including incorrect information or inadequate information in their books. I think this version of discussion is clearer to those looking for improved editions of the related books used in Nepal.

We removed the discussion about first aid and oriented book authors and prospective first aiders and teachers to refer to the "World Health Organization 2016. Guidelines for the management of snakebites as well as other national guidelines and scientific documentations.

To shorten the lengthy discussion, we removed other less important texts from the discussion section. These texts were:

"This issue of persistently adherence towards substandard or harmful first aid should be a priority of education and health systems of the associated nations to ensure adequately edited books being used in formal education. Further, periodic evaluation of the educational materials used in formal education of medical and non-medical students and the vulnerable community people about safe and effective first aid is essential in preventing further harm to snakebite patients. It is because the"

"Further, harmful practices adopted by caregiver of snake-bitten-children being significantly associated with low maternal education and low paternal education [4] highlighted the need of formal education of snakebite at schools and universities in Nepal and elsewhere. Hence, the origin of these errors should be investigated to help eliminating erroneous mentions about first aid in the textbooks and consequential wrong first aid for snakebite at communities."

### Reviewer #5:

Not given any comments.

#### Author responses:

Thank you so much for the next intensive review and approval of this section.

# B] Editorial and Data Presentation Modifications?

Use this section for editorial suggestions as well as relatively minor modifications of existing data that would enhance clarity. If the only modifications needed are minor and/or editorial, you may wish to recommend “Minor Revision” or “Accept”.

## Reviewer #1:

None

#### Author responses:

Thank you.

## Reviewer #5:

Accept.

#### Author responses:

Thank you so much for the next intensive review and approval of this manuscript.

# C] Summary and General Comments

Use this section to provide overall comments, discuss strengths/weaknesses of the study, novelty, significance, general execution and scholarship. You may also include additional comments for the author, including concerns about dual publication, research ethics, or publication ethics. If requesting major revision, please articulate the new experiments that are needed.

## Reviewer #1:

The authors have adequately addressed most of the issues highlighted at the first review.

#### Author responses:

Thank you so much for the next intensive review.

## Reviewer #5:

Given comments addressed and improved.

#### Author responses:

Thank you so much for the next intensive review.

# D] PLOS authors have the option to publish the peer review history of their article

([what does this mean?](https://track.editorialmanager.com/CL0/https:%2F%2Fjournals.plos.org%2Fplosntds%2Fs%2Feditorial-and-peer-review-process%23loc-peer-review-history/1/010f01997af88c98-ba180a64-b0af-4b82-b771-f5108db35e01-000000/uNIJQV019xVdKuKu3s62s1hUBDJqQf0mNGnVtxC85GQ=230)). If published, this will include your full peer review and any attached files.
If you choose “no”, your identity will remain anonymous but your review may still be made public.
**Do you want your identity to be public for this peer review?** For information about this choice, including consent withdrawal, please see our [Privacy Policy](https://track.editorialmanager.com/CL0/https:%2F%2Fwww.plos.org%2Fprivacy-policy/1/010f01997af88c98-ba180a64-b0af-4b82-b771-f5108db35e01-000000/vqAJpWt7iQ83Oqa9K8h4G9sLHbUi3l-UT6Tb2Hu7uEQ=230).

## Reviewer #1:

No

## Reviewer #5:

No

#### Author responses:

Thank you both of you reviewers.

# E] [NOTE:

If reviewer comments were submitted as an attachment file, they will be attached to this email and accessible via the submission site. Please log into your account, locate the manuscript record, and check for the action link "View Attachments". If this link does not appear, there are no attachment files.]

#### Author responses:

We downloaded the review comments from reviewer 5, for which we appreciate the reviewer 5.

# F] Figure resubmission:

While revising your submission, we strongly recommend that you use PLOS’s NAAS tool (<https://ngplosjournals.pagemajik.ai/artanalysis>) to test your figure files. NAAS can convert your figure files to the TIFF file type and meet basic requirements (such as print size, resolution), or provide you with a report on issues that do not meet our requirements and that NAAS cannot fix.

After uploading your figures to PLOS’s NAAS tool -<https://ngplosjournals.pagemajik.ai/artanalysis>, NAAS will process the files provided and display the results in the "Uploaded Files" section of the page as the processing is complete. If the uploaded figures meet our requirements (or NAAS is able to fix the files to meet our requirements), the figure will be marked as "fixed" above. If NAAS is unable to fix the files, a red "failed" label will appear above. When NAAS has confirmed that the figure files meet our requirements, please download the file via the download option, and include these NAAS processed figure files when submitting your revised manuscript.

#### Author responses:

We uploaded Figures 1-3, Supplementary Figures 1-2, and Box 1 that are fixed by NAAS tool.

# G] Reproducibility:

To enhance the reproducibility of your results, we recommend that authors of applicable studies deposit laboratory protocols in [protocols.io](http://protocols.io/), where a protocol can be assigned its own identifier (DOI) such that it can be cited independently in the future. Additionally, PLOS ONE offers an option to publish peer-reviewed clinical study protocols. Read more information on sharing protocols at <https://plos.org/protocols?utm_medium=editorial-email&utm_source=authorletters&utm_campaign=protocols>

#### Author responses:

We do not have protocols to submit.

# References/evidence used while responding to the reviewers

1. Bollig G, Myklebust AG, Østringen K (2011) Effects of first aid training in the kindergarten- a pilot study. Scandinavian Journal of Trauma, Resuscitation and Emergency Medicine 19: 13.

2. Zdenek CN, Rodrigues CF, Bourke LA, Tanaka-Azevedo AM, Monagle P, et al. (2023) Children and snakebite: snake venom effects on adult and paediatric plasma. Toxins 15: 158.

3. Guile L, Lee A, Gutiérrez JM (2023) Factors associated with mortality after snakebite envenoming in children: a scoping review. Transactions of the Royal Society of Tropical Medicine and Hygiene 117: 617–627.

4. Dayasiri K, Suraweera N, Burhan P (2025) First-aid practices and pre-hospital care in paediatric snakebites. BMC Pediatrics 25: 614.
